# Gastric acid suppression promotes alcoholic liver disease by inducing overgrowth of intestinal *Enterococcus*

**DOI:** 10.1038/s41467-017-00796-x

**Published:** 2017-10-16

**Authors:** Cristina Llorente, Peter Jepsen, Tatsuo Inamine, Lirui Wang, Sena Bluemel, Hui J. Wang, Rohit Loomba, Jasmohan S. Bajaj, Mitchell L. Schubert, Masoumeh Sikaroodi, Patrick M. Gillevet, Jun Xu, Tatiana Kisseleva, Samuel B. Ho, Jessica DePew, Xin Du, Henrik T. Sørensen, Hendrik Vilstrup, Karen E. Nelson, David A. Brenner, Derrick E. Fouts, Bernd Schnabl

**Affiliations:** 1 Department of Medicine, University of California San Diego, La Jolla, CA 92093 USA; 2 Department of Medicine, VA San Diego Healthcare System, San Diego, CA 92161 USA; 3 Department of Hepatology and Gastroenterology, Aarhus University Hospital, Aarhus, 8000 Denmark; 4 Department of Clinical Epidemiology, Aarhus University Hospital, Aarhus, 8000 Denmark; 5 Department of Pharmacotherapeutics, Nagasaki University Graduate School of Biomedical Sciences, Nagasaki, 852-8523 Japan; 6 Division of Gastroenterology, Hepatology and Nutrition, Virginia Commonwealth University and McGuire VA Medical Center, Richmond, VA 23249 USA; 7 Microbiome Analysis Center, George Mason University, Manassas, VA 20110 USA; 8 Department of Surgery, University of California San Diego, La Jolla, CA 92093 USA; 9 J. Craig Venter Institute, Rockville, MD 20850 USA

## Abstract

Chronic liver disease is rising in western countries and liver cirrhosis is the 12th leading cause of death worldwide. Simultaneously, use of gastric acid suppressive medications is increasing. Here, we show that proton pump inhibitors promote progression of alcoholic liver disease, non-alcoholic fatty liver disease, and non-alcoholic steatohepatitis in mice by increasing numbers of intestinal *Enterococcus* spp. Translocating enterococci lead to hepatic inflammation and hepatocyte death. Expansion of intestinal *Enterococcus faecalis* is sufficient to exacerbate ethanol-induced liver disease in mice. Proton pump inhibitor use increases the risk of developing alcoholic liver disease among alcohol-dependent patients. Reduction of gastric acid secretion therefore appears to promote overgrowth of intestinal *Enterococcus*, which promotes liver disease, based on data from mouse models and humans. Recent increases in the use of gastric acid-suppressive medications might contribute to the increasing incidence of chronic liver disease.

## Abstract (web summary)

Proton pump inhibitors (PPIs) reduce gastric acid secretion and modulate gut microbiota composition. Here Llorente *et al*. show that PPIs induce bacterial overgrowth of enterococci, which, in turn, exacerbate ethanol-induced liver disease both in mice and humans.

## Introduction

The number of people with chronic liver disease is increasing rapidly in western countries. Liver cirrhosis as end-stage organ disease is now the 12th leading cause of death worldwide^1^. The increase is partly due to the increasing prevalence of obesity, which is associated with non-alcoholic fatty liver disease (NAFLD) and steatohepatitis (NASH)^2^. Approximately 50% of all cirrhosis-associated deaths are related to alcohol^3^.

Proton pump inhibitors (PPIs), which reduce gastric acid secretion, are among the most commonly prescribed medications in the world. There has been a substantial growth in the total PPI use, and approximately 6–15% of the general population is receiving acid suppression therapy^4, 5^; 32% of patients with NAFLD^6^ and 67–72% of patients with cirrhosis take acid-reducing medications^7, 8^. Gastric acid kills ingested microbes, and suppression of gastric acid secretion can change the composition of the intestinal microbiota^9^.

We investigated the effects of gastric acid suppression on progression of chronic liver disease. Here we report that gastric acid suppression induces overgrowth of intestinal *Enterococcus* and its translocation to the liver. In the liver, hepatic macrophages and Kupffer cells recognize *Enterococcus* and induce interleukin-1 beta (IL1B) secretion contributing to ethanol-induced liver inflammation and hepatocyte damage. We provide evidence from mice and humans that gastric acid suppression promotes liver injury and progression of chronic liver disease.

## Results

### Absence of gastric acid exacerbates alcohol-induced liver disease

**Fig. 1:**
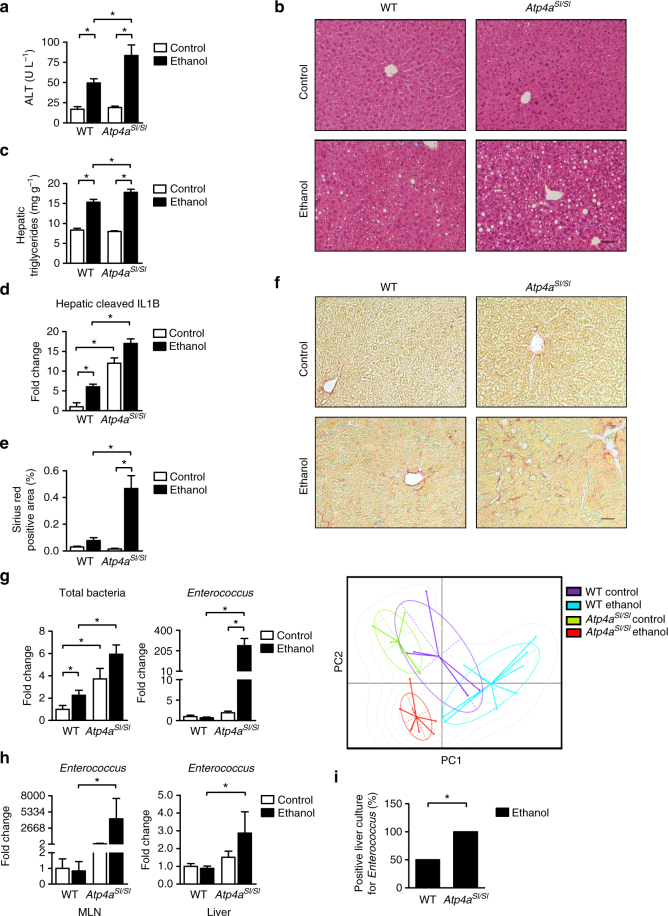
Genetic deletion of gastric acid secretion exacerbates alcohol-induced liver disease in mice. **a**–**h** WT mice and their *Atp4a*^*Sl/Sl*^ littermates were fed an oral control diet (*n* = 3–8; 2–3 replicates) or ethanol diet (*n* = 6–16; 8–9 replicates) for 9 weeks following the chronic Lieber DeCarli diet model. **a** Plasma levels of ALT. **b** Representative liver sections after hematoxylin and eosin staining. **c** Hepatic triglyceride content. **d** Hepatic expression of cleaved IL1B protein (*n* = 2–5). **e** Hepatic areas of fibrosis were identified by staining with Sirius red (*n* = 2–6); area was quantitated by image analysis software. **f** Representative Sirius red-stained liver sections. **g** Total bacteria in feces (*left panel*). Fecal enterococci (*mid panel*). Principal component analysis of fecal microbiomes, performed using the *ade4* R package^46^ (*right panel*). **h** *Enterococcus* in mesenteric lymph nodes (MLN) and liver, assessed by qPCR. **i** Proportions of positive *Enterococcus* cultures from liver tissues of WT mice (*n* = 6) and their *Atp4a*^*Sl/Sl*^ littermates (*n* = 11) subjected to chronic-plus-binge ethanol feeding. Results are expressed as mean ± s.e.m. *Scale bars* = 100 μm. For **a**, **c**, **d**, **e**, **g**, **h** significance was evaluated using the unpaired Student *t*-test or Mann–Whitney *U*-statistic test. For **i**, significance was evaluated using Fisher’s exact test.*P < 0.05

We first determined the role of gastric acid on ethanol-induced liver disease in *Atp4a*^*Sl/Sl*^ mice, which have a point mutation in *Atp4a* (the gene encoding the gastric H^+^, K^+^-ATPase α subunit) and develop achlorhydria (absent gastric acid)^10^. *Atp4a*^*Sl/Sl*^ mice developed more severe ethanol-associated liver disease than littermates with wild-type *Atp4a* (WT). Following ethanol administration, the *Atp4a*^*Sl/Sl*^ mice showed more severe liver injury, based on level of alanine aminotransferase (ALT) and hepatic steatosis, than WT mice (Fig. 1a–c and Supplementary Fig. 1a–c).

In *Atp4a*^*Sl/Sl*^ mice, liver disease progressed from simple steatosis to steatohepatitis. Inflammation was identified based on the hepatic increase in levels of the macrophage marker F4/80 (indicating more inflammatory Kupffer cells; Supplementary Fig. 1d, e), de novo expression of the *Ccl2* and *Cxcl5* genes, which encode inflammatory chemokines (Supplementary Fig. 1f), and higher levels of active (cleaved) IL1B protein (Fig. 1d and Supplementary Fig. 1g). In addition, livers from ethanol-fed *Atp4a*^*Sl/Sl*^ mice became fibrotic (Fig. 1e, f) and had increased staining for smooth muscle α-actin (ACTA2), a marker of activated myofibroblasts and stellate cells, which contribute to the development of fibrosis (Supplementary Fig. 1h, i). Absence of gastric acid (due to the mutation in *Atp4a* in mice) did not affect intestinal absorption or hepatic metabolism of ethanol (Supplementary Fig. 1j, k).

Chronic administration of ethanol is associated with intestinal bacterial overgrowth and dysbiosis^11^. To determine whether the absence of gastric acid altered the composition of the intestinal microbiota, luminal bacteria were measured by quantitative PCR (qPCR), and changes in the microbiota were analyzed by 16S ribosomal RNA (rRNA) gene sequencing. Ethanol administration resulted in intestinal bacterial overgrowth and dysbiosis in both strains of mice, but levels of these were increased to a significantly greater extent in *Atp4a*^*Sl/Sl*^ mice than in WT mice (Fig. 1g). One of the most prominent changes identified by 16S rRNA sequencing was an increased proportion of *Enterococcus* spp. (Gram-positive cocci) in the microbiota of *Atp4a*^*Sl/Sl*^ mice compared with WT mice after ethanol feeding (Supplementary Fig. 2a), which was confirmed by qPCR (Fig. 1g). We measured proportions of *Escherichia coli* (*E. coli*) and *Prevotella* spp. (both Gram-negative rods) as controls. The proportion of *E. coli* increased by a non-significant amount in *Atp4a*^*Sl/Sl*^ mice fed ethanol compared to WT mice fed ethanol. On the other hand, the proportion of *Prevotella* was significantly reduced in *Atp4a*^*Sl/SI*^ mice fed ethanol compared with WT mice fed ethanol (Supplementary Fig. 2a).

Development of alcoholic liver disease (ALD) involves increased translocation of microbial products from the intestinal lumen to the liver, facilitated by disruption of the intestinal epithelial barrier^12^. Following ethanol administration, paracellular intestinal permeability (as quantified by detection of albumin in the feces) and plasma level of endotoxin (lipopolysaccharide, LPS) increased to similar levels in WT and *Atp4a*^*Sl/Sl*^ mice (Supplementary Fig. 2b). To relate changes in the microbiome to translocation, enterococci were measured in extra-intestinal tissues. Numbers of gut-derived and translocated *Enterococcus* were significantly higher in mesenteric lymph nodes and liver tissues of *Atp4a*^*Sl/Sl*^ than WT mice following chronic ethanol administration as measured by qPCR (Fig. 1h). There was no significant difference in the amount of *E. coli* and *Prevotella* translocated to mesenteric lymph nodes and liver between *Atp4a*^*Sl/Sl*^ and WT mice following chronic ethanol administration (Supplementary Fig. 2c). A significantly higher proportion of bacterial cultures from liver tissues of *Atp4a*^*Sl/Sl*^ mice given ethanol were positive for *Enterococcus* than from WT mice given ethanol in a second model of alcoholic liver disease (Fig. 1i). *Atp4a*^*Sl/Sl*^ mice were confirmed to have more severe ethanol-associated liver disease in this chronic-plus-binge model^13^ (Supplementary Fig. 3a–f), consistent with the chronic Lieber DeCarli model. These results indicate that ethanol feeding promotes specific expansion of intestinal *Enterococcus* and its translocation to the liver in the absence of gastric acid.

### NAFLD is increased in the absence of gastric acid secretion

**Fig. 2:**
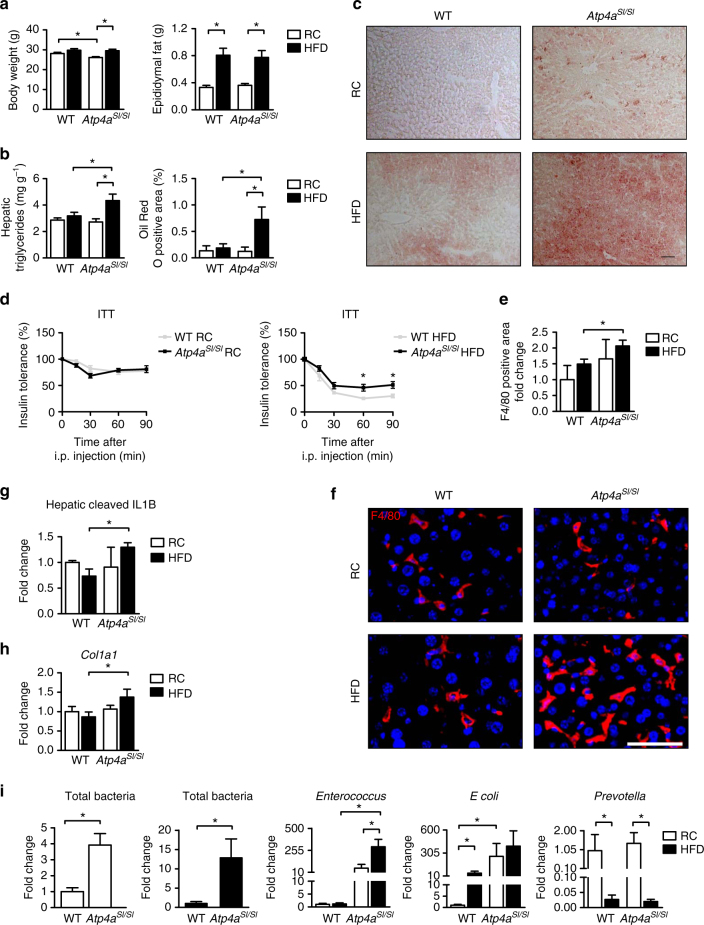
NAFLD is increased in *Atp4a*^*Sl/Sl*^ mice. WT mice and their *Atp4a*^*Sl/Sl*^ littermates were fed a regular chow (RC) diet (*n* = 5–8; 2–3 replicates) or a HFD (*n* = 6–15; 3–6 replicates) for 9 weeks. **a** Body weight and weight of white epididymal fat. **b** Hepatic triglyceride content and hepatic steatosis visualized with Oil Red O staining and quantified by image analysis software (*n* = 5–10). *Scale bar* = 100 μm. **c** Representative Oil Red O-stained liver sections. **d** Insulin tolerance test (ITT) (*n* = 5–10). **e**–**f** Representative liver sections of F4/80 immunofluorescence staining; the positively stained area was quantified by image analysis software (*n* = 3–6). *Scale bar* = 50 μm. **g** Hepatic levels of cleaved IL1B (*n* = 2–5). **h** Hepatic expression of mRNA encoding *Col1a1*. **i** Total bacteria, *Enterococcus*, *E. coli*, and *Prevotella* in fecal samples, measured by qPCR. Changes in fecal numbers of *E. coli* and *Prevotella* did not differ significantly between *Atp4a*^*Sl/Sl*^ mice fed a HFD vs. WT mice fed a HFD (*n* = 5–10). *Atp4a*^*Sl/Sl*^ mice fed a HFD had significantly higher numbers in *Enterococcus* than WT mice fed a HFD. Results are expressed as mean ± s.e.m. For **a**, **b**, **d**, **e**, **g**, **h**, **i** significance was evaluated using the unpaired Student *t*-test or Mann–Whitney *U*-statistic test. *P < 0.05

We extended our study to mice with metabolic liver diseases. A high-fat diet (HFD) induces NAFLD in mice. *Atp4a*^*Sl/Sl*^ mice fed a HFD for 9 weeks did not differ from WT mice fed a HFD in body weight or weight of white adipose tissue (Fig. 2a). However, hepatic steatosis was more severe (Fig. 2b, c) and insulin sensitivity was reduced in *Atp4a*^*Sl/Sl*^ mice on a HFD compared to WT mice on a HFD (Fig. 2d). A higher number of liver macrophages and increased levels of active IL1B were observed in *Atp4a*^*Sl/Sl*^ mice on a HFD (Fig. 2e–g). Hepatic expression of the *Col1a1* gene was induced in *Atp4a*^*Sl/Sl*^ mice on a HFD, compared with WT mice on a HFD (Fig. 2h). Similar to the ethanol-induced liver disease, there was a strong increase in bacterial overgrowth and the proportion of *Enterococcus* in the microbiota of *Atp4a*^*Sl/Sl*^ mice fed a HFD, compared with WT mice fed a HFD (Fig. 2i).

### Absence of gastric acid secretion exacerbates NASH

**Fig. 3:**
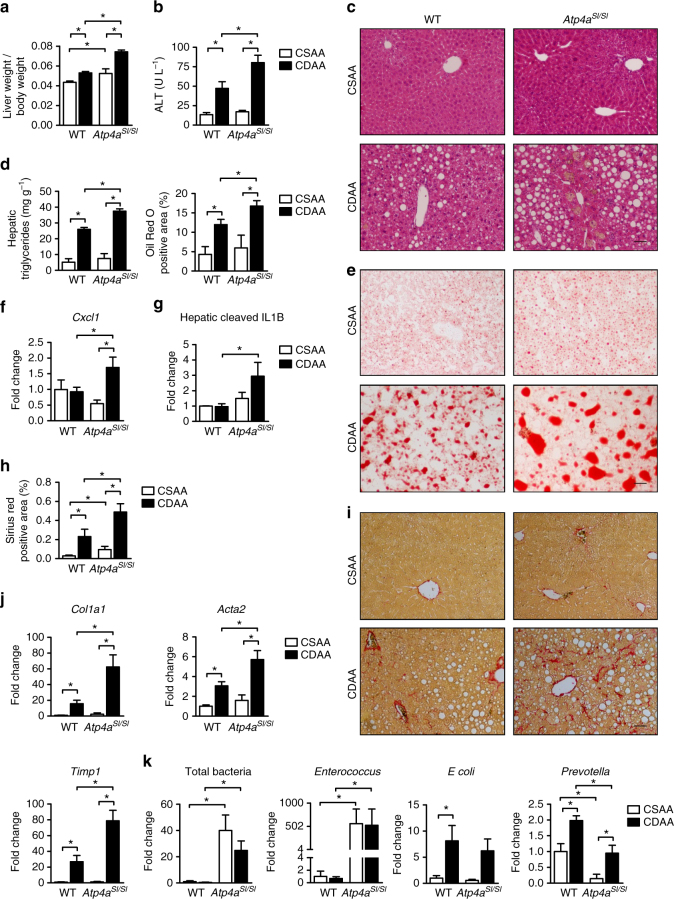
Exacerbated NASH in *Atp4a*^*Sl/Sl*^ mice. WT mice and their *Atp4a*^*Sl/Sl*^ littermates were fed a CSAA (control, *n* = 4–9; 1–3 replicates) or CDAA diet (*n* = 10–12; 4–5 replicates) for 20 weeks. **a** Ratio of liver to body weight was significantly higher in CDAA-fed *Atp4a*^*Sl/Sl*^ mice than CDAA-fed WT mice, **b** as was mean plasma level of ALT. **c** Representative liver sections, stained with hematoxylin and eosin. **d** Hepatic triglyceride content. The Oil Red O-stained area was quantified by image analysis (*n* = 5–12). **e** Representative Oil Red O-stained liver sections. **f** Hepatic expression of mRNA encoding the chemokine *Cxcl1*. **g** Hepatic levels of cleaved IL1B (*n* = 2–5). **h** Collagen deposition was evaluated by Sirius red staining and quantified by image analysis (*n* = 5–13). **i** Representative sections stained with Sirius red. **j** Hepatic expression of genes involved in liver fibrosis including *Col1a1*, *Acta2* (smooth muscle α-actin, a marker of activated myofibroblasts), and *Timp1* (tissue inhibitor of metalloproteinase 1). **k** Total bacteria, proportions of *Enterococcus*, *E. coli*, and *Prevotella* in fecal samples, measured by qPCR. Proportions of fecal *E. coli* did not differ significantly between WT and *Atp4a*^*Sl/Sl*^ mice. Numbers of *Prevotella* were lower in *Atp4a*^*Sl/Sl*^ mice than in WT mice, with or without CDAA feeding. Numbers of *Enterococcus* were significantly higher in *Atp4a*^*Sl/Sl*^ mice than in WT mice, with or without CDAA feeding. *Scale bars* = 100 μm. Results are expressed as mean ± s.e.m. For **a**, **b**, **d**, **f**, **g**, **h**, **j**, **k** significance was evaluated using the unpaired Student *t*-test or Mann–Whitney *U*-statistic test.*P < 0.05

Mice were fed a choline-deficient l-amino acid-defined (CDAA) diet for 20 weeks to induce histologic changes that resemble those observed in patients with NASH. Absence of gastric acid exacerbated NASH (*Atp4a*^*Sl/Sl*^ vs. WT mice), demonstrated by increased liver to body weight ratio (Fig. 3a), levels of ALT (Fig. 3b), steatosis (Fig. 3c–e), inflammation (Fig. 3f, g), and fibrosis (Fig. 3h–j). Total numbers of intestinal bacteria and numbers of *Enterococcus* were significantly higher in *Atp4a*^*Sl/Sl*^ mice than WT mice, with and without the CDAA diet (Fig. 3k). Taken together, disruption of *Atp4a*, which controls gastric acid secretion, increases the severity of alcohol-induced liver disease, NAFLD, and NASH in mice.

### PPIs promote progression of ethanol-induced steatohepatitis

**Fig. 4:**
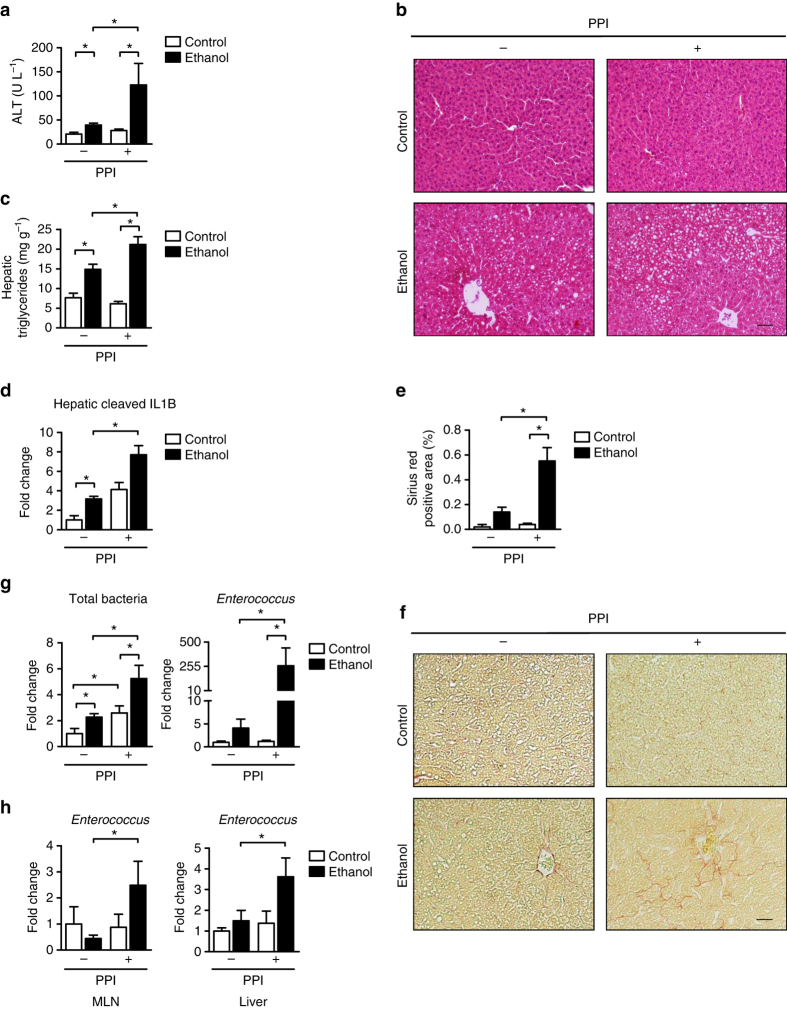
Pharmacological gastric acid suppression promotes progression of alcohol-induced liver disease in mice. C57BL/6 mice were fed an oral control diet (*n* = 4–6; 1–2 replicates) or ethanol diet (*n* = 11–18; 1–2 replicates) that contained PPI (200 p.p.m.) or vehicle (water) for 9 weeks. **a** Plasma levels of ALT. **b** Representative liver sections after hematoxylin and eosin staining. **c** Hepatic triglyceride content. **d** Hepatic levels of cleaved IL1B protein (*n* = 2–5). **e** Hepatic areas of fibrosis were identified by staining with Sirius red (*n* = 2–7); area was quantitated by image analysis software. **f** Representative Sirius red-stained liver sections. **g** Total bacteria and total amount of enterococci in feces. **h** *Enterococcus* in mesenteric lymph nodes (MLN) and liver, assessed by qPCR. *Scale bars* = 100 μm. Results are expressed as mean ± s.e.m. For **a**, **c**–**e**, **g**, **h** significance was evaluated using the unpaired Student *t*-test or Mann–Whitney *U*-statistic test. *P < 0.05

We performed pharmacologic studies with PPIs (omeprazole) to confirm our findings from the genetic model of achlorhydria. Mice were given doses of PPI similar to those of previous studies^14^, which increased gastric pH to that of the *Atp4a*^*Sl/Sl*^ mice^10^ (Supplementary Fig. 4a). This PPI dose was higher than that usually given to patients, because the PPI administered to mice lacks enteric coating and undergoes rapid degradation in the stomach^15^. Consistent with our data from *Atp4a*^*Sl/Sl*^ mice, C57BL/6 mice receiving a PPI developed more severe ethanol-induced liver injury, steatosis, inflammation, and fibrosis than mice not receiving a PPI (Fig. 4a–f and Supplementary Fig. 4b–j). The PPI did not affect absorption or hepatic metabolism of ethanol (Supplementary Fig. 5a, b).

PPI administration was associated with significant increases in numbers of fecal bacteria and *Enterococcus* following ethanol administration (Fig. 4g and Supplementary Fig. 5c). Suppression of gastric acid resulted in differences of the spatial distribution of intestinal enterococci—significantly higher numbers of *Enterococcus* associated with the mucosa of the small intestine following ethanol administration (Supplementary Fig. 5d). Impaired control of the mucosa-associated microbiota leads to increased bacterial translocation and facilitates progression of alcoholic liver disease^16^. Translocation of *Enterococcus* to mesenteric lymph nodes and the liver was increased in ethanol-fed mice also given a PPI, compared with those that did not receive a PPI (Fig. 4h and Supplementary Fig. 5e).

### Dynamics of intestinal *Enterococcus* growth

**Fig. 5:**
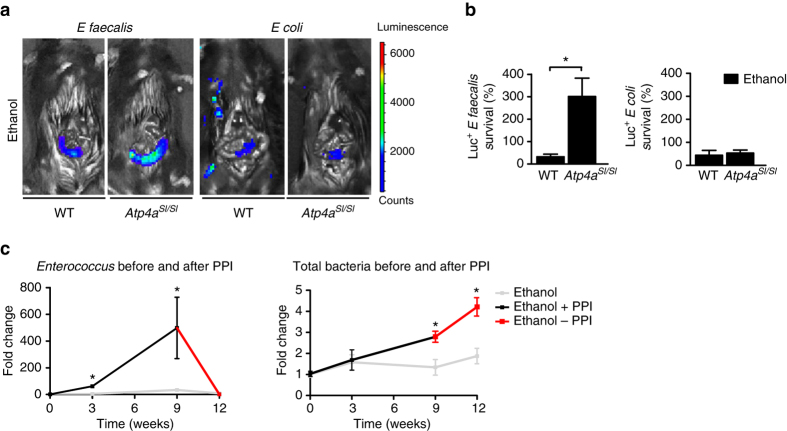
Changes of intestinal *Enterococcus* growth in the absence of gastric acid during chronic ethanol feeding. **a** WT mice and their *Atp4a*^*Sl/Sl*^ littermates were fed an ethanol diet (*n* = 3–5) for 2 weeks. We used an in vivo assay to measure luminal killing of *E. coli* and *E. faecalis* in the gut^17, 38, 42^. A 4 cm loop of the proximal jejunum was ligated (without interrupting the blood supply) in anesthetized mice and injected with bioluminescent *E. coli* or *E. faecalis*. To analyze luminal survival and killing, we performed IVIS imaging of bioluminescent *E. coli* and *E. faecalis* at 80 and 90 min, respectively, after injection of bacteria into ligated jejunal loops. Representative images are shown. Whereas loops of ethanol-fed *Atp4a*^*Sl/Sl*^ mice had a similar amount of bioluminescent *E. coli* than ethanol-fed WT mice, bioluminescent *E. faecalis* was found to be ninefold higher in *Atp4a*^*Sl/Sl*^ than WT mice. **b** The *graph* shows survival in percentage of injected *E. coli* and *E. faecalis*. **c** C57BL/6 mice were fed an ethanol diet with or without a PPI (200 p.p.m.) for 9 weeks, before the PPI was discontinued. Fecal *Enterococcus* and total luminal bacteria were measured by qPCR (*n* = 2–12) (2 replicates). Results are expressed as mean ± s.e.m. For **b**, **c** significance was evaluated using the unpaired Student *t-*test or Mann–Whitney *U*-statistic test. *P < 0.05

We used an in vivo assay to measure killing of bioluminescent bacteria in ligated jejunal loops^17^. A larger number of *Enterococcus faecalis* (*E. faecalis*) survived and proliferated in the jejunum in the absence of gastric acid (*Atp4a*^*Sl/Sl*^ mice) than in WT mice; this difference was not observed for *E. coli* (controls; Fig. 5a, b). This result indicates that *E. faecalis* favors a less acidic environment, compared to *E. coli*, during ethanol feeding. The increase in intestinal *Enterococcus* after increases in gastric pH is rapid and reversible—numbers of enterococci decreased back to baseline levels after PPIs were withdrawn for 3 weeks. In contrast, the total numbers of intestinal bacteria remained elevated, due to the continual presence of ethanol (Fig. 5c).

**Fig. 6:**
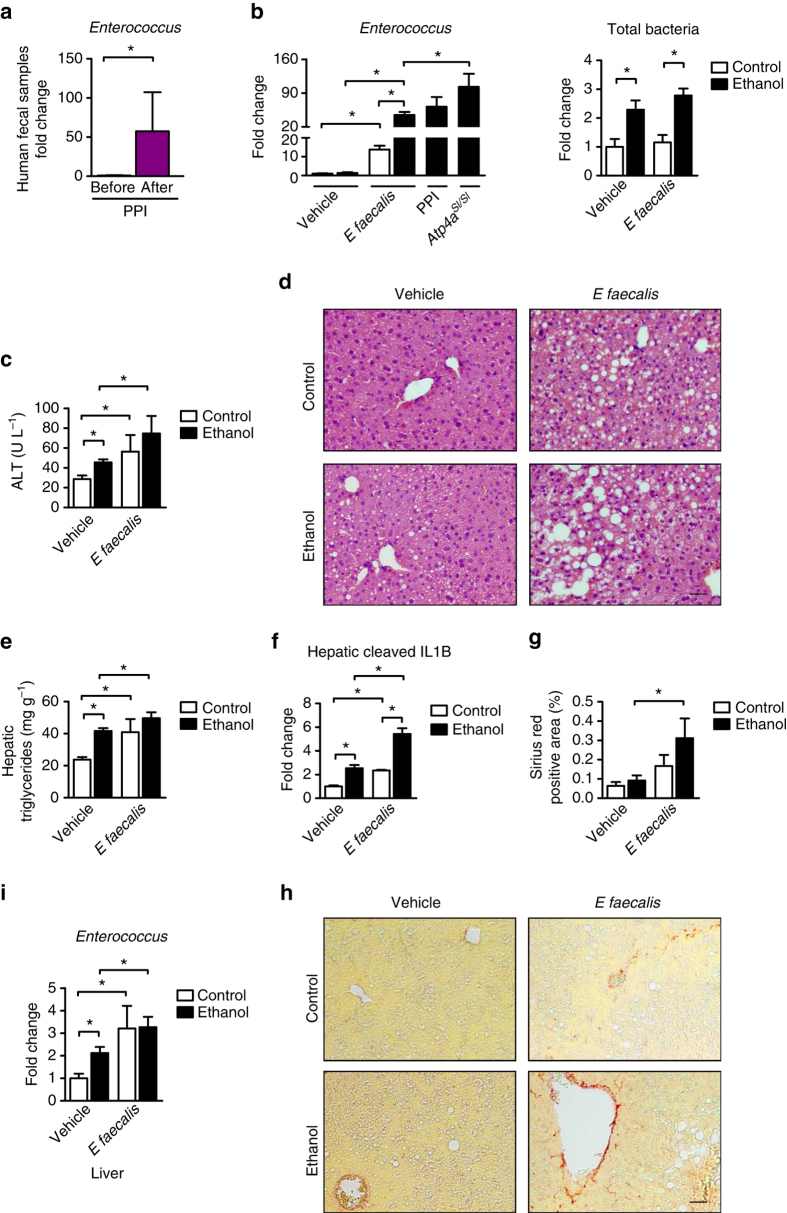
Numbers of *Enterococcus* in human fecal samples and effects of *E. faecalis* on progression of alcohol-induced liver disease in mice. **a** Fecal amounts of *Enterococcus* in healthy individuals before (*n* = 13) and after taking a PPI (omeprazole, 40 mg) daily for 14 days (*n* = 12). **b**–**i** C57BL/6 mice were gavaged with polymyxin B 150 mg kg^−1^ and neomycin 200 mg kg^−1^ body weight once daily for 1 week to facilitate colonization of *E. faecalis*. Mice were then fed an oral control diet (*n* = 3–9; 1–2 replicates) or ethanol diet (*n* = 13–25; 1–2 replicates) for 9 weeks and gavaged with *E. faecalis* (5 × 10^9^ CFUs) or vehicle (water) every third day. **b** Enterococci (*left panel*) and total amount of bacteria (*right panel*) in feces. Samples used to measure enterococci in ethanol-fed *Atp4a*^*Sl/Sl*^ mice and in C57BL/6 mice fed ethanol or given PPIs are the same as in Figs. 1g and 2g, respectively. **c** Plasma levels of ALT. **d** Representative liver sections after hematoxylin and eosin staining. **e** Hepatic triglyceride content. **f** Hepatic expression of cleaved IL1B protein (*n* = 2–5). **g** Hepatic areas of fibrosis were identified by staining with Sirius red (*n* = 3–10); area was quantitated by image analysis software. **h** Representative Sirius red-stained liver sections. **i** *Enterococcus* in liver, assessed by qPCR. *Scale bars* = 100 μm. Results are expressed as mean ± s.e.m. For **a**–**c**, **e**–**g**, **i** significance was evaluated using the unpaired Student *t*-test or Mann–Whitney *U*-statistic test. *P < 0.05

To extend our preclinical findings to humans, enterococci were measured in fecal samples collected from healthy individuals before and after PPI (omeprazole) therapy. Numbers of *Enterococcus* significantly increased in samples collected after 2 weeks of PPI treatment vs. before (Fig. 6a).

### *E. faecalis* enhances ethanol-induced liver disease

We then tested whether overgrowth of intestinal *Enterococcus* is sufficient to increase alcohol-induced liver disease. We have recently shown that a complete absence of the microbiota exacerbates acute ethanol-induced liver disease in germ-free mice. At baseline, germ-free mice have an altered xenobiotic response to drugs and increased hepatic ethanol metabolism^18^. Gnotobiotic mice are therefore not an ideal disease model to manipulate the intestinal microbiota during ethanol-induced liver disease. To mimic longer lasting overgrowth of intestinal enterococci following gastric acid suppression, overgrowth was induced by repeated gavage of C57BL/6 mice with *E. faecalis*, which was isolated from feces of an ethanol-fed *Atp4a*^*Sl/Sl*^ mouse. *E. faecalis* was selected, because it was among the three most abundant *Enterococcus* spp. in intestines of ethanol-fed *Atp4a*^*Sl/Sl*^ mice, as identified by 16S rRNA sequencing (Supplementary Fig. 2a).

Numbers of *Enterococcus* in feces from gavaged and ethanol-fed mice increased significantly, similar to the increase observed after genetic or pharmacologic reduction of gastric acid (Fig. 6b). Colonization of mice with *E. faecalis* did not affect the total number of bacteria following chronic ethanol administration (Fig. 6b). Therefore, experimental expansion of intestinal *E. faecalis* during alcohol feeding mimics alcohol-induced alterations of the microbiota in mice with suppressed gastric acid secretion, and represents a good model to study the role of *E. faecalis* for ethanol-induced liver disease. Increasing intestinal numbers of *E. faecalis* led to translocation of enterococci and exacerbated ethanol-induced liver injury, steatosis, inflammation, and fibrosis in mice. Mild liver disease was induced by *E. faecalis* in control mice that did not receive ethanol (Fig. 6c–i and Supplementary Fig. 6a–j). These findings indicate that *Enterococcus* promotes progression of chronic liver disease in mice.

### Mechanism of *E. faecalis* -exacerbated alcoholic liver disease

To further define the mechanism by which *Enterococcus* increases liver disease, we generated Toll-like receptor 2 (TLR2), or myeloid differentiation primary response 88 (MYD88)/TIR-domain-containing adapter-inducing interferon-β (TRIF; also known as TICAM1) bone-marrow chimeric mice using a combination of clodronate-mediated Kupffer cell depletion, irradiation, and bone-marrow transplantation. WT mice were given bone marrow transplants from WT, *Tlr2*^−/−^, or *Myd88*^−/−^/*Trif*^*LPS2/LPS2*^ mice, which results in full reconstitution of Kupffer cells^19, 20^.

**Fig. 7:**
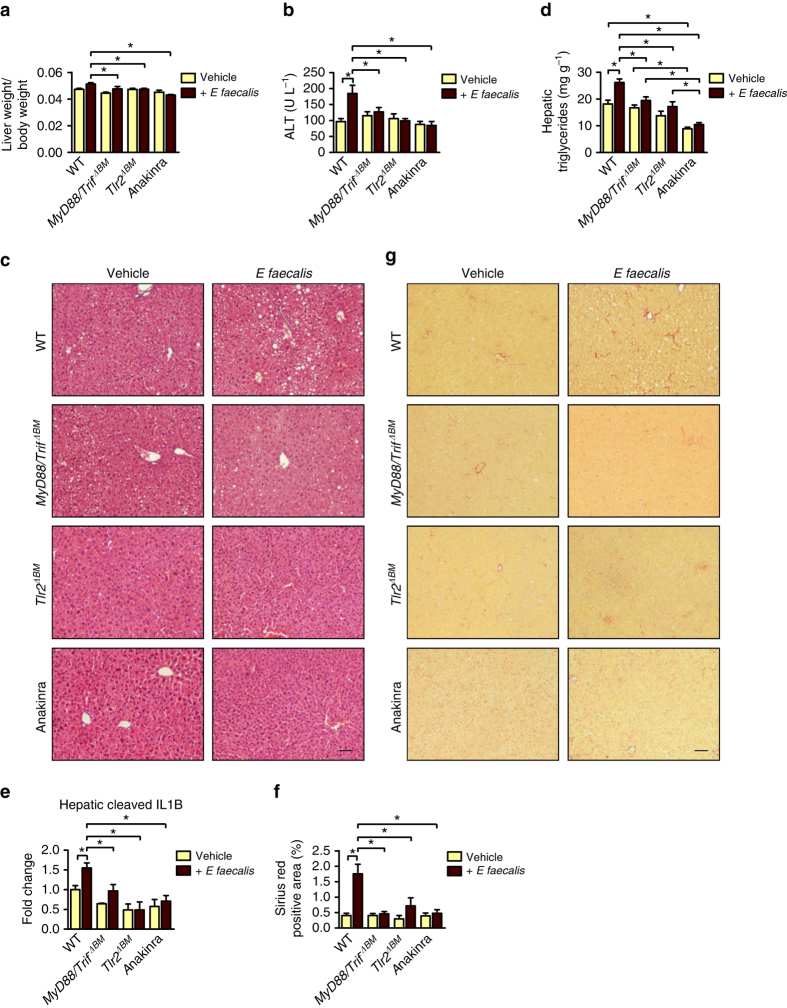
MYD88/TRIF, TLR2 or IL1-receptor inhibition protects from *E. faecalis* -exacerbated alcoholic liver disease. C57BL/6 mice were irradiated, given transplants of WT, *Myd88*^−/−^*/Trif*^*LPS2/LPS2*^ (*Myd88/Trif*^*ΔBM*^), or *Tlr2*^−/−^ bone marrow (*Tlr2*^*ΔBM*^) and injected with clodronate liposomes. Mice were then gavaged with polymyxin B 150 mg kg^−1^ and neomycin 200 mg kg^−1^ body weight once daily for 1 week to facilitate colonization of *E. faecalis*, fed the ethanol diet for 9 weeks, and gavaged with *E. faecalis* (5 × 10^9^ CFUs; *n* = 14–27; 1–3 replicates) or vehicle (water; *n* = 14–29) (1–3 replicates) every third day. A subset of WT mice given transplants of WT bone marrow received the IL1-receptor antagonist anakinra. **a** Ratio of liver to body weight. **b** Plasma levels of ALT. **c** Representative liver sections after hematoxylin and eosin staining. **d** Hepatic triglyceride content. **e** Hepatic levels of cleaved IL1B (*n* = 3). **f** Hepatic areas of fibrosis were identified by staining with Sirius red; area was quantified by image analysis software (*n* = 6–17). **g** Representative Sirius red-stained liver sections. *Scale bar* = 100 μm. Results are expressed as mean ± s.e.m. For **a**, **b**, **d**–**f** significance was evaluated using one-way analysis of variance with Newman–Keuls post-test. *P < 0.05

TLR2 is a cell membrane receptor that recognizes products from Gram-positive bacteria such as peptidoglycan^21^. MYD88 and TRIF are intracellular adaptor molecules for pathogen recognition receptors such as TLRs; mice that do not express MYD88 and TRIF lack innate immune signaling^22, 23^. Chimeric mice with Kupffer cells that do not express MYD88/TRIF or TLR2 were protected from *E. faecalis* -exacerbated alcoholic liver injury (Fig. 7a, b), steatosis (Fig. 7c, d), inflammation (Fig. 7e), and fibrosis (Fig. 7f, g), compared with chimeric mice with WT Kupffer cells. Intestinal absorption or hepatic metabolism of ethanol was not affected in chimeric mice (Supplementary Fig. 7a, b).

Interestingly, despite being protected from *E. faecalis* -exacerbated alcoholic liver disease, chimeric mice with Kupffer cells that did not express MYD88/TRIF had a significantly higher percentage of positive *Enterococcus* blood cultures than mice with WT or *Tlr2*^−/−^ Kupffer cells (Supplementary Fig. 7c). This indicates that viable *Enterococcus* reaches the liver and mediates ethanol-induced liver disease via binding to TLR2 on Kupffer cells and induction of hepatic inflammation. Under normal circumstances, viable bacteria are cleared efficiently by Kupffer cells, which prevents prolonged exposure of microbes to pathogen recognition receptors. Phagocytosis is impaired in the absence of MYD88 and TRIF in Kupffer cells by mechanisms that deserve future investigations.

**Fig. 8:**
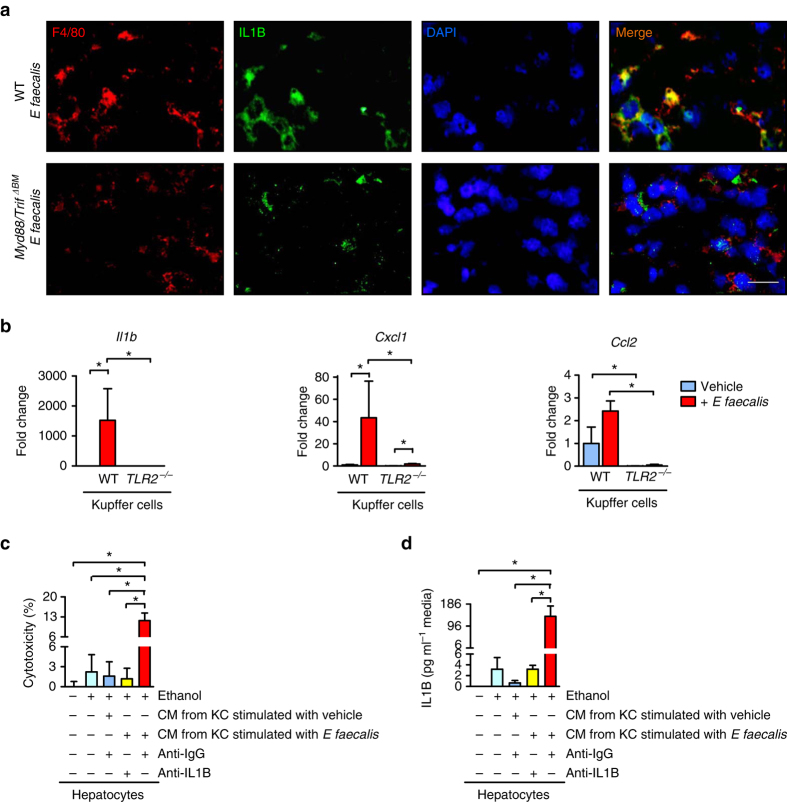
*E. faecalis* causes an inflammatory and hepatotoxic response mediated by TLR2 on Kupffer cells. **a** C57BL/6 mice were irradiated, given transplants of WT or Myd88^−/−^/Trif^LPS2/LPS2^ bone marrow (*Myd88/Trif*^*ΔBM*^), and injected with clodronate liposomes. Mice were then gavaged with polymyxin B 150 mg kg^−1^ and neomycin 200 mg kg^−1^ body weight once daily for 1 week to facilitate colonization of *E. faecalis*, fed the ethanol diet for 9 weeks and gavaged with *E. faecalis* (5 × 10^9^ CFUs) every third day. Immunofluorescence analysis of F4/80 (*red*) and IL1B (*green*; representative liver sections); nuclei are blue. *Scale bar* = 10 μm. **b** Primary mouse WT and *Tlr2*^−/−^ Kupffer cells were stimulated with heat-inactivated, sonicated *E. faecalis* for 8 h; expression of genes encoding inflammatory mediators was measured (*n* = 4–5 independent experiments). Results are expressed relative to the level of unstimulated WT Kupffer cells within each experiment. **c**, **d** Conditioned medium (CM) from Kupffer cells (KC; stimulated or not stimulated with heat-inactivated, sonicated *E. faecalis*) was transferred to ethanol-stimulated (100 mM) primary mouse hepatocytes in the presence of a control (IgG) or IL1B neutralizing antibody. **c** Hepatocyte cytotoxicity (*n* = 3 independent experiments performed in 2–6 replicates) and total IL1B in the cell supernatant (2–6 replicates) (**d**). Results are expressed as mean ± s.e.m.. For **b**–**d** significance was evaluated using the unpaired Student *t*-test or Mann–Whitney *U*-statistic test. *P < 0.05

IL1B mediates alcoholic steatohepatitis in mice^24^. Levels of hepatic IL1B protein significantly increased after *E. faecalis* expansion and administration of ethanol. This increase was blocked in mice with Kupffer cells that did not express MYD88/TRIF or TLR2 (Fig. 7e). Using immunofluorescence analyses, we found that in livers of ethanol-fed mice, F4/80 co-localized with IL1B (Fig. 8a). We therefore isolated primary Kupffer cells from WT and *Tlr2*^−/−^ mice and stimulated with inactivated *E. faecalis*. WT, but not *Tlr2*^−/−^ Kupffer cells, increased gene expression of inflammatory mediators, such as *Il1b*, *Cxcl1*, and *Ccl2* (Fig. 8b). Incubation of ethanol-primed mouse hepatocytes with conditioned medium from *E. faecalis* -stimulated Kupffer cells increased hepatocyte cytotoxicity in the presence of an isotype control antibody. This cytotoxic effect was blocked with a neutralizing antibody against IL1B (Fig. 8c). Cytotoxicity correlated with the secreted amount of total IL1B in the supernatant (Fig. 8d).

These results indicate that translocated *Enterococcus* binds to TLR2 on Kupffer cells to increase IL1B secretion and liver cell damage. To further demonstrate that ILB, as downstream target of TLR2, mediates *E. faecalis* -exacerbated alcoholic liver disease in vivo, mice were treated with the ILB receptor antagonist anakinra. Anakinra-treated mice were protected from *E. faecalis* -exacerbated alcoholic liver disease (Fig. 7 and Supplementary Fig. 7).

### PPIs increase the risk of liver disease in chronic alcoholics

**Fig. 9:**
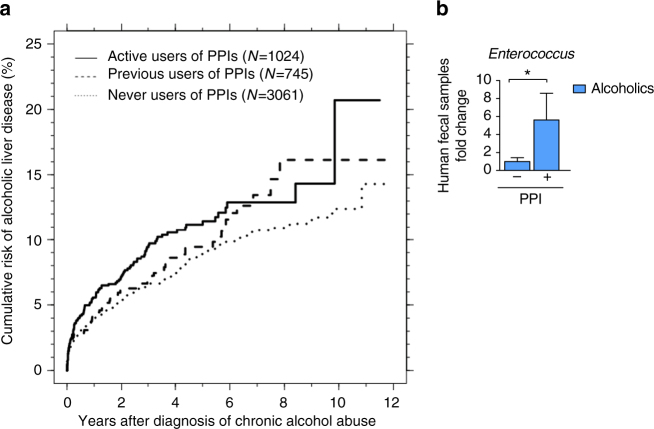
PPI treatment increases the risk of liver disease in chronic alcoholics. **a** Cumulative risk of ALD based on use of PPIs. A Cox regression model was used to compare risk among active, previous, and never users. **b** Fecal amounts of *Enterococcus* in chronic consumers of alcohol taking PPIs (*n* = 3) and not taking PPI (*n* = 8). Results are expressed as mean ± s.e.m. For **a**, a Cox regression model was used to compare risk among PPI users in a cohort of alcohol-dependent patients.For **b**, significance was evaluated using Student *t-*test. *P < 0.05

We next examined the association between use of PPIs and development of ALD among chronic alcohol abusers. Of 4830 patients with a diagnosis of chronic alcohol abuse, 1024 (21%) were active users of PPIs, 745 (15%) were previous users, and 3061 (63%) had never used PPIs. The 3 groups were similar with respect to demographics and liver-related biochemistry at inclusion (Supplementary Table 1). The 10-year risk of a diagnosis of ALD was 20.7% for active users of PPIs, 16.1% for previous users, and 12.4% for never users (Fig. 9a). The active users had a significantly higher risk of developing ALD than previous users (adjusted hazard ratio (HR) for active users vs. previous users = 1.37; 95% confidence interval (CI), 1.00–1.88) or never-users (adjusted HR for active users vs. never users = 1.52; 95% CI, 1.21–1.91). We did not find any confounding variables that could account for these associations. Finally, we observed significantly greater numbers of *Enterococcus* in fecal samples from patients who abuse alcohol and use PPI concomitantly than patients who abuse alcohol and do not use PPIs (Fig. 9b).

## Discussion

**Fig. 10:**
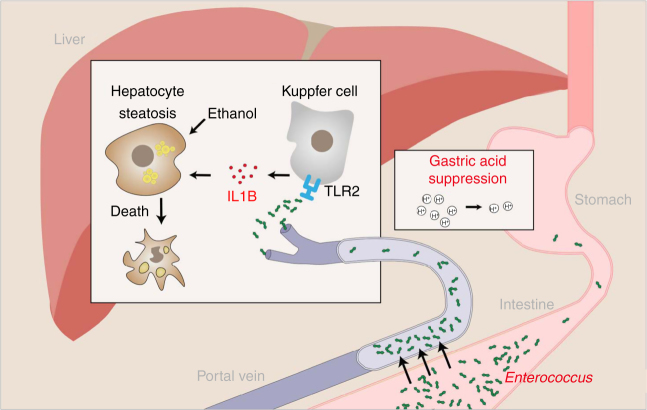
Gastric acid suppression and alcoholic liver disease. Gastric acid suppression increases intestinal *Enterococcus*, which translocates to the liver via the portal vein. *Enterococcus* binds to the pathogen recognition receptor TLR2 on hepatic Kupffer cells, leading to secretion of IL1B. IL1B contributes to ethanol-induced liver inflammation and hepatocyte damage

Changes in the gastrointestinal homeostasis can promote liver disease^25, 26^. Our findings link an increase in *Enterococcus* with induction of hepatic inflammation, via the pathogen-recognition receptor TLR2, and progression of liver disease (Fig. 10). Virulence factors of *Enterococcus*, such as gelatinase E^27^, might facilitate bacterial translocation and could also contribute to liver disease. *Enterococcus* has also been found to cause spontaneous bacterial peritonitis in patients with end-stage liver disease. In patients with cirrhosis, risk of bacterial infections and their complications is strongly associated with acid suppressive medication^28^. Hence, the side effects of gastric acid suppression are not limited to development and progression of pre-cirrhotic liver disease, but also include infections commonly observed in patients with cirrhosis.

Importantly, we demonstrate that in alcohol-dependent patients, gastric acid suppression promotes the onset and progression of liver disease. Although a randomized study is required to confirm data from our cohort study, our findings indicate that the recent rise in use of gastric acid-suppressing medications might have contributed to the increased incidence of chronic liver disease. In our cohort, 36% of alcohol-dependent patients have been using PPIs. Although obesity and alcohol use predispose to acid reflux requiring antacid medication, many patients with chronic liver disease take gastric acid suppressive medications without appropriate indication^29^. Clinicians should consider withholding medications that suppress gastric acid unless there is a strong medical indication.

## Methods

### Mice

Sublytic *Atp4a*^*Sl/Sl*^ mice generated on a hybrid background of 129/SvJ and Black Swiss strains, and backcrossed to the C57BL/6 J background. *Atp4a*^*Sl/Sl*^ mice were created by using *N*-ethyl*-N*-nitrosourea to induce a causal mutation (T → C transition) in *Atp4a*, encoding the α subunit of the gastric H^+^,K^+^-ATPase^10^. Heterozygous *Atp4a*^*+/Sl*^ mice on a C57BL/6 genetic background were used for breeding, and mice with unmutated *Atp4a* (WT) and *Atp4a*^*Sl/Sl*^ littermates were used in experiments. C57BL/6 mice were purchased from Charles River and used in PPI, *E. faecalis*, and bone-marrow transplantation studies.

Female mice (age, 10 weeks) were used in Lieber DeCarli diet model experiments for 9 weeks^30^. In brief, the Lieber DeCarli diet comprises Micro Stabilized Rod Liq AC IRR (LD101A; TestDiet), Maltodextrin IRR (9598; TestDiet) and 200-proof ethanol (Koptec). The caloric intake from ethanol was 0 on day 1, 10% of total calories on days 2 and 3, 20% on days 4 and 5, 30% from day 6 until the end of 6 weeks, and 36% for the last 3 weeks. Control mice received an isocaloric amount of iso-maltose instead of ethanol. For the chronic-plus-binge ethanol feeding model^13^, age-matched female mice were fed the Lieber DeCarli diet for 15 days followed by an ethanol binge. The caloric intake from ethanol was 0 on days 1–5 and 36% from day 6 until the end. At day 16, mice were gavaged with a single dose of ethanol (5 g kg^−1^ body weight) in the early morning and then sacrificed 9 h later. Mice were pair-fed and the amount of liquid diet containing ethanol was similar between mouse strains within each experiment (Supplementary Fig. 8a–e).

To induce non-alcoholic fatty liver disease, male mice (age 10 weeks) were fed a high-fat diet (HFD; 59% energy from fat; S3282; Bio-Serv) for 9 weeks. HFD intake was not different between WT and *Atp4a*^*Sl/Sl*^ mice (Supplementary Fig. 8f). A choline-deficient L-amino acid-defined (CDAA) diet (518753; Dyets) was given for 20 weeks to induce non-alcoholic steatohepatitis. A choline-supplemented L-amino acid-defined (CSAA) diet (518754; Dyets) served as control diet. CDAA diet intake did not differ between WT and *Atp4a*^*Sl/Sl*^ mice (Supplementary Fig. 8g).

Omeprazole (Fagron) was mixed, at indicated doses, into liquid diets. To reduce intestinal bacteria, female C57BL/6 (Charles River) mice were gavaged with polymyxin B (McKesson) 150 mg kg^−1^ and neomycin (Sigma-Aldrich) 200 mg kg^−1^ body weight once daily for 1 week. Following eradication of the commensal microbiota, mice were gavaged with 5 × 10^9^ CFUs *E. faecalis* (or water as control) every third day. *E. faecalis* was isolated from an ethanol-fed *Atp4a*^*Sl/Sl*^ mouse. The identity was confirmed by 16S rRNA PCR (see below) and sequence analyses. *E. faecalis* was grown freshly in Bacto Brain-Heart infusion medium (Becton Dickinson) for each gavage.

As described, *Atp4a*^*Sl/Sl*^ mice develop iron deficiency anemia at ages of 4–6 weeks. To replace iron, WT and *Atp4a*^*Sl/Sl*^ littermate mice were placed on a high-iron diet (containing 2% carbonyl iron diet; 7012, Envigo) for 4 weeks^10^. Mice were then maintained on a regular chow diet (RC; 5053; LabDiet) for 1 week before experiments. Four weeks of iron supplementation reversed anemia and iron deficiency for the length of all experimental procedures (9 weeks for alcohol-induced liver disease, Supplementary Fig. 8h; 9 weeks for feeding of a HFD, Supplementary Fig. 8j; 20 weeks for CDAA-induced steatohepatitis; Supplementary Fig. 8k). Total plasma and liver levels of iron did not differ significantly between ethanol-fed WT and *Atp4a*^*Sl/Sl*^ mice at the end of the treatment period (Supplementary Fig. 8i).

For bone marrow transplantation, C57BL/6 recipient mice were given lethal doses of radiation (650 rads) twice, using a ^137^Cs source. Two weeks after bone marrow transplantation, mice were given intraperitoneal injections of 200 μl of clodronate liposomes (5 mg ml^−1^; Vrije Universiteit, The Netherlands) to deplete radio-resistant Kupffer cells. The Lieber DeCarli diet began 4 weeks after bone marrow transplantation. C57BL/6 mice and mice deficient in MYD88 and TRIF (*Myd88*^−/−^*/Trif*^*LPS2/LPS2*^)^22, 23^ or TLR2 (Jackson laboratory) on a C57BL/6 genetic background were used as bone marrow donors.

To block IL1 signaling, mice were given intraperitoneal injections of anakinra (Amgen; 25 mg kg^−1^ daily) for the last 5 weeks of the experiment.

All animal studies were reviewed and approved by the Institutional Animal Care and Use Committee of the University of California, San Diego.

### Bacterial DNA isolation and 16S rRNA sequencing

DNA was isolated from feces, liver, or mesenteric lymph nodes of mice. Samples were resuspended in PBS and digested with RNAse A and proteinase K at 55 °C for one hour. Each suspension was then transferred to individual Qbiogene lysing matrix B tubes and vortexed using a FastPrep FP120 instrument. The lysate was then extracted twice using Phenol/Chloroform/Isoamyl alcohol, precipitated and washed with ethanol, and the DNA resuspended in TE buffer^11, 31, 32^. Genomic DNA was isolated from the mucus layer from a 2 cm piece of the proximal small intestine (jejunum). The exact length, width, and weight of this piece were measured. Luminal contents were collected by flushing with 1 ml sterile PBS. The remaining intestine was cut longitudinally and washed vigorously in 1 ml PBS to collect the mucus and its associated bacteria^16^. We performed deep DNA pyrosequencing of fecal DNA targeting the hypervariable V1–V3 region of prokaryotic 16S rRNA loci using 454 GS FLX Titanium technology to generate microbial community profiles using species level (97% similarity) operational taxonomic unit-based classification and analysis, as previously described^11, 32^. Sequence data were registered at NCBI under BioProject PRJNA294003. Sequence reads are available at NCBI under the following consecutive BioSample IDs: SAMN04032754-SAMN04032783.

### *Enterococcus* cultures

Blood and liver were collected in a sterile fashion. Liver was homogenized using a beads beater, and liquid enterococcus cultures were incubated for 72 h at 37 °C under anaerobic conditions in a selective medium, BBL Enterococosel broth (Becton Dickinson). Positive cultures were identified by the brown-black color generated by the hydrolysis of esculin to esculetin that reacts with ferric citrate.

### Real-time qPCR

RNA was extracted from mouse tissues and cDNAs were generated^20^. Primer sequences for mouse genes were obtained from the NIH qPrimerDepot. To quantify the total bacterial load present in feces, the qPCR value of 16 S rRNA gene for each sample was multiplied by the total amount of DNA (μg) per mg of feces. The following published bacterial primer sequences were used: 16 S rRNA gene^33^, *Enterococcus*^34^, *E. coli*^35^, and *Prevotella*^36^. The qPCR value of bacteria was normalized to the total amount of 16S. To quantify enterococci in mesenteric lymph nodes or liver, the qPCR value of *Enterococcus* for each sample was multiplied by the total amount of DNA (μg) per mg of tissue. Genomic bacterial DNA was extracted from the liver, and the qPCR value of bacteria was normalized to the host 18S gene or total amount of DNA (μg) per mg of liver. Mouse gene expression and amplification of genomic bacterial DNA were determined with Sybr Green (Bio-Rad Laboratories) using the ABI StepOnePlus real-time PCR system. All gene expression data were expressed relative to control-fed WT mice.

### In vivo luminal killing assay

Non-pathogenic *E. coli* were transformed with the pXen13 plasmid (Caliper)—a vector carrying the original *Photorhabdus luminescens* luxCDABE operon for engineering bioluminescent bacteria. This operon is not expressed in Gram-positive bacteria. Therefore for expression in *E. faecalis* (isolated from an ethanol-fed *Atp4a*^*Sl/Sl*^ mouse), pSL101P_16s_ harboring the luxABCDE cassette under the control of a synthetic *E. faecalis* 16S RNA P_1_ promoter^37^ (provided by Dag Anders Brede, Norwegian University of Life Sciences, Ås, Norway) was used. To assess the killing and survival rate of non-pathogenic and bioluminescent bacteria in vivo, we used an intestinal loop model^17, 38^. After anesthesia, a midline laparotomy incision was made. A segment of the jejunum approximate 4 cm long was created with two vascular hemoclips without disrupting the mesenteric vascular arcades. The length of intestine between the two clips was injected with 5.7 × 10^5^ CFUs. Bioluminescence imaging was performed using IVIS Spectrum (Caliper) per instructions of the manufacturer. Mice were kept anaesthetized and bioluminescence was recorded between 80 and 90 min after injection of bioluminescent bacteria.

### Biochemical analysis

Plasma levels of ethanol were measured using the Ethanol Assay Kit (BioVision). Levels of ALT were measured using Infinity ALT kit (Thermo Scientific). Hepatic triglyceride levels were measured using the Triglyceride Liquid Reagents Kit (Pointe Scientific). Hepatic alcohol dehydrogenase (ADH) activity was measured using the ADH Assay Kit (BioVision). Plasma LPS (Cloud-Clone Corp), and fecal albumin levels (Bethyl lab) were measured by ELISA. Whole blood was collected into potassium EDTA–containing Microvette 100 tubes (Sarstedt), and automated complete blood counts (CBCs) were obtained using a Scil Vet abc automatic hematology analyzer (Scil Animal Care). For insulin tolerance test (ITT), following a 6-hour fasting period, an intraperitoneal dose of 0.35 or 1 U kg^−1^ insulin (novolin, Novo Nordisk Inc.) was administered to mice on RC diet or HFD, respectively, and blood glucose was measured at indicated time points. Plasma and liver levels of iron were measured using a kit from Thermo Scientific.

### Measurement of gastric acid

Gastric pH was measured as described^39, 40^. In brief, gastroesophageal and gastroduodenal junctions were ligated and total gastrectomy was performed. Saline (500 μl) was injected into the lumen and the whole stomach was placed in an oxygenated bath (37 °C) containing HEPES buffer (pH 7.4). Histamine (Thermo Fisher Scientific; 200 μM) was added to the bath for 60 min, the injected non-buffered saline solution was aspirated from the stomach, and the pH was measured with a pH probe (Accumet AB15 Basic and BioBasic; Thermo Fisher Scientific).

### Staining procedures

Formalin-fixed tissue samples were embedded in paraffin (Paraplast plus, McCornick) and stained with hematoxylin-eosin (Surgipath). For hepatic lipid accumulation analysis, 5 μm frozen sections were cut and stained with Oil Red O (Sigma-Aldrich). Hepatic fibrosis was assessed by morphometric analysis of the Sirius red-stained area. For Sirius Red staining, liver tissues were fixed in 10% buffered formalin, embedded in paraffin and sectioned at 5 μm thickness. Sections were stained with Sirius Red solution (saturated picric acid (Sigma-Aldrich)) containing 0.1% Direct Red 80 (Sigma-Aldrich) to visualize collagen deposition^41^. F4/80, 1:50 (14-4801-82; eBioscience), ACTA2, 1:100 (ab5694; Abcam) and IL1B, 1:50 (ab9722; Abcam) immunofluorescence staining was performed using frozen section for the double staining with IL1B and F4/80 staining or paraffin embedded sections for F4/80 or ACTA staining^16^. All samples were analyzed by densitometry, using NIH Image J. Formalin-fixed tissue samples were used for double-immunofluorescence analyses.

### Immunoblot analyses

To measure expression of cytochrome P450 family 2 subfamily E polypeptide 1 (CYP2E1), mouse liver microsomes were isolated as described^42^. Immunoblot analysis was performed using anti-CYP2E, 1:1000 (AB1252; Millipore Corporation). Antibodies against VDAC1, 1:5000 (ab14734; Abcam) and β-actin 1:5000 (A5441; Sigma) were used to ensure equal loading for microsome and whole-tissue extracts, respectively. An antibody against IL1B, 1:1000 (ab9722; Abcam) was used to detect the cleaved (active) form of ILB (17 kDa). Immunoblots were visualized with a charged coupling device camera in a luminescent image analyzer (Gel-Doc; Bio-Rad). Immunoblots were analyzed by densitometry, using Science Lab 2001 Image Gauge version 4.0, FUJIFILM. Uncropped immunoblots are shown in Supplementary Figs. 9−12.

### Cell culture experiments

For primary mouse hepatocytes isolation, livers were first perfused in situ with 0.5 mM EGTA containing calcium-free salt solution, followed by perfusion with solution containing 0.02% (w v^−1^) collagenase D (Roche Applied Science). The liver was then gently minced on a Petri dish and filtered using a 70 μm nylon cell strainer. Hepatocytes were washed three times and centrifuged at 50 × *g* for 1 min. Cell viability was consistently 85% as determined by Trypan Blue (Thermo Fisher Scientific) exclusion. A total of 1.5 × 10^5^ cells were plated on 12-well plates coated with rat collagen type I in DMEM-F12 (Thermo Fisher Scientific) with insulin-transferrin-selenium (1% v v^−1^) (Thermo Fisher Scientific) and 40 ng ml^−1^ dexamethasone (MP Biomedicals) containing 10% (v v^−1^) fetal bovine serum (FBS; Gemini Bio-Products) and antibiotics. After 4 h, the culture was washed with DMEM-F12 media and changed to the same complemented media without FBS^41^.

Next day, primary mouse Kupffer cells were isolated from mice by two-step collagenase–pronase perfusion followed by three-layer discontinuous density gradient centrifugation with 8.2% (w v^−1^) and 14.5% (w v^−1^) Nycodenz (Accurate Chemical and Scientific Corporation) to obtain Kupffer-cell fraction. Kupffer cell fraction was selected positively by magnetic cell sorting using anti-CD11b Micro Beads (Miltenyi Biotech). 2 × 10^5^ Kupffer cells were plated on 12-well plates and cultured with RPMI 1640 (Thermo Fisher Scientific) containing 10% (v v^−1^) FBS for 4 h. Following an overnight starvation in medium without FBS, Kupffer cells were stimulated with heat-inactivated and sonicated *E. faecalis* (MOI 25) for 8 h, respectively. RNA was extracted from Kupffer cells and used for qPCR.

For conditioned medium and IL1B neutralization experiments, Kupffer cells were cultured with RPMI 1640 medium containing 10% FBS (v v^−1^) for 4 h and serum starved in DMEM/F-12 (Thermo Fisher Scientific) with insulin-transferrin-selenium (1% v v^−1^) (Thermo Fisher Scientific) and 40 ng ml^−1^ dexamethasone (MP Biomedicals) overnight. Kupffer cells were then stimulated with heat-inactivated and sonicated *E. faecalis* (MOI 25) for 8 hours with the same culture medium containing 10% FBS (v v^−1^). Cell supernatants were transferred to hepatocytes, and incubated for 24 h. A neutralizing antibody against IL1B (10 ng ml^−1^; ab9722; Abcam) or isotype IgG (10 ng ml^−1^; Santa Cruz Biotechnologyl) was added to the conditioned medium after the transfer. Hepatocyte cytotoxicity was assessed using the Pierce LDH Cytotoxicity Detection Kit (Thermo Fisher Scientific), and total IL1B was measured in the cell supernatant by ELISA (eBioscience).

### Human stool samples

Healthy individuals without chronic disease who were not taking PPIs (controls, *n* = 14) were given omeprazole (40 mg) to be taken daily before breakfast for 14 days. Baseline features of this cohort have been described by us^9^. Stool samples were collected before and after 14 days of PPI treatment. DNA extracted from fecal samples was used for qPCR to detect *Enterococcus*, and normalized to 16S rRNA concentrations. One sample collected before PPI administration and two collected afterward had too low DNA concentrations and could not be amplified by qPCR. Written informed consent was signed by each participant after the nature and possible consequences of the studies were explained. The study protocol was approved by the Institutional Review Board from each institution involved.

Alcohol-dependent patients taking PPI (*n* = 3) or not taking PPI (*n* = 8) were included in the study. All patients were actively drinking. Stool samples were collected and RNA was extracted from fecal samples^32^ and RT-qPCR performed. Written informed consent was signed by each participant after the nature and possible consequences of the studies were explained. The study protocol was approved by the Institutional Review Board from each institution involved.

### Human study population

We used data from Danish population based healthcare databases covering a population region with 2.2 million inhabitants to examine the association between PPI use and development of ALD among chronic alcohol abusers. Chronic alcohol abusers were identified through the Danish National Patient Registry, which contains hospital discharge diagnoses from every inpatient or outpatient hospital contact, coded according to ICD-10^43^. Hospital diagnoses of ALD were identified through the same registry (ICD-10: K70.x), and PPI use was identified through the pharmacy registry of reimbursed prescriptions covering all pharmacies in 1998 or later^44^. Results of liver biochemistry tests were extracted from the regional laboratory information system covering all hospitals.

We followed all patients with a first-time diagnosis of chronic alcohol abuse after 1 January 2001 (ICD-10 codes: F10.x except F10.0) from their initial diagnosis to the date they were diagnosed with ALD (ICD-10: K70.x) or died. Other patients were censored on 1 January 2013. We excluded patients who did not receive all of the following liver-related tests during the period from 90 days before until 7 days after their first diagnosis of chronic alcohol abuse: bilirubin, INR, creatinine, albumin, ALT, platelet count, and sodium. We used the cumulative incidence function to compute the cumulative risk of being diagnosed with ALD for groups defined by PPI history up to inclusion (never user, previous user, or active user)^45^. We defined previous users as those who had not filled a prescription for PPI in the year before study inclusion, but who had filled a prescription prior to that year. Diagnoses of alcoholic liver disease were made based on the combination of history of alcohol abuse and increased levels of liver enzymes, in the absence of other causes for liver disease. We used Cox regression to compare rates of diagnosis of ALD among the 3 groups, adjusting for confounding by sex, age, and liver-related biochemistry at the time of inclusion. The Danish Data Protection Board has approved the study (record no 2013-41-1924).

### Statistical analysis

Results are expressed as mean ± s.e.m. Numbers for biological replicates are listed in Supplementary Data 1. Significance was evaluated using the unpaired Student *t* test, Mann–Whitney *U*-statistic test, or one-way analysis of variance with Newman–Keuls post-test. Fisher’s exact test was used in the analysis of positive *Enterococcus* cultures. A Cox regression model was used to compare risk among PPI users in a cohort of alcohol-dependent patients. A *P* value < 0.05 was considered to be statistically significant.

### Data availability

The data that support the findings of this study are available from the authors on reasonable request. The mouse gut metagenome was deposited under NCBI BioProject PRJNA294003 and sequence reads are available at NCBI under the following consecutive BioSample IDs: SAMN04032754-SAMN04032783.

## Electronic supplementary material

Supplementary Information

Supplementary Data 1
