# Publisher Correction: Gastric acid suppression promotes alcoholic liver disease by inducing overgrowth of intestinal *Enterococcus*

**DOI:** 10.1038/s41467-017-01779-8

**Published:** 2017-12-12

**Authors:** Cristina Llorente, Peter Jepsen, Tatsuo Inamine, Lirui Wang, Sena Bluemel, Hui J. Wang, Rohit Loomba, Jasmohan S. Bajaj, Mitchell L. Schubert, Masoumeh Sikaroodi, Patrick M. Gillevet, Jun Xu, Tatiana Kisseleva, Samuel B. Ho, Jessica DePew, Xin Du, Henrik T. Sørensen, Hendrik Vilstrup, Karen E. Nelson, David A. Brenner, Derrick E. Fouts, Bernd Schnabl

**Affiliations:** 1 Department of Medicine, University of California San Diego, La Jolla, CA 92093 USA; 2 Department of Medicine, VA San Diego Healthcare System, San Diego, CA 92161 USA; 3 Department of Hepatology and Gastroenterology, Aarhus University Hospital, Aarhus, 8000 Denmark; 4 Department of Clinical Epidemiology, Aarhus University Hospital, Aarhus, 8000 Denmark; 5 Department of Pharmacotherapeutics, Nagasaki University Graduate School of Biomedical Sciences, Nagasaki, 852-8523 Japan; 6 Division of Gastroenterology, Hepatology and Nutrition, Virginia Commonwealth University and McGuire VA Medical Center, Richmond, VA 23249 USA; 7 Microbiome Analysis Center, George Mason University, Manassas, VA 20110 USA; 8 Department of Surgery, University of California San Diego, La Jolla, CA 92093 USA; 9 J. Craig Venter Institute, Rockville, MD 20850 USA

*Nature Communications* **8**:837 10.1038/s41467-017-00796-x; Article published online: 16 Oct 2017

In the original PDF version of this Article, which was published on 16 October 2017, the publication date was incorrectly given as 10 October 2017. This has now been corrected in the PDF; the HTML version of the paper was correct from the time of publication.

