# Supplementary material for: Gastric acid suppression promotes alcoholic liver disease by inducing overgrowth of intestinal Enterococcus
Source: Nat Commun. 2017 Oct 16;8:837. doi: 10.1038/s41467-017-00796-x (PMC5643518; doi:10.1038/s41467-017-00796-x)
Supplement: Supplementary file 1 — Supplementary Information [file 41467_2017_796_MOESM1_ESM.pdf]

Supplementary Figure 1

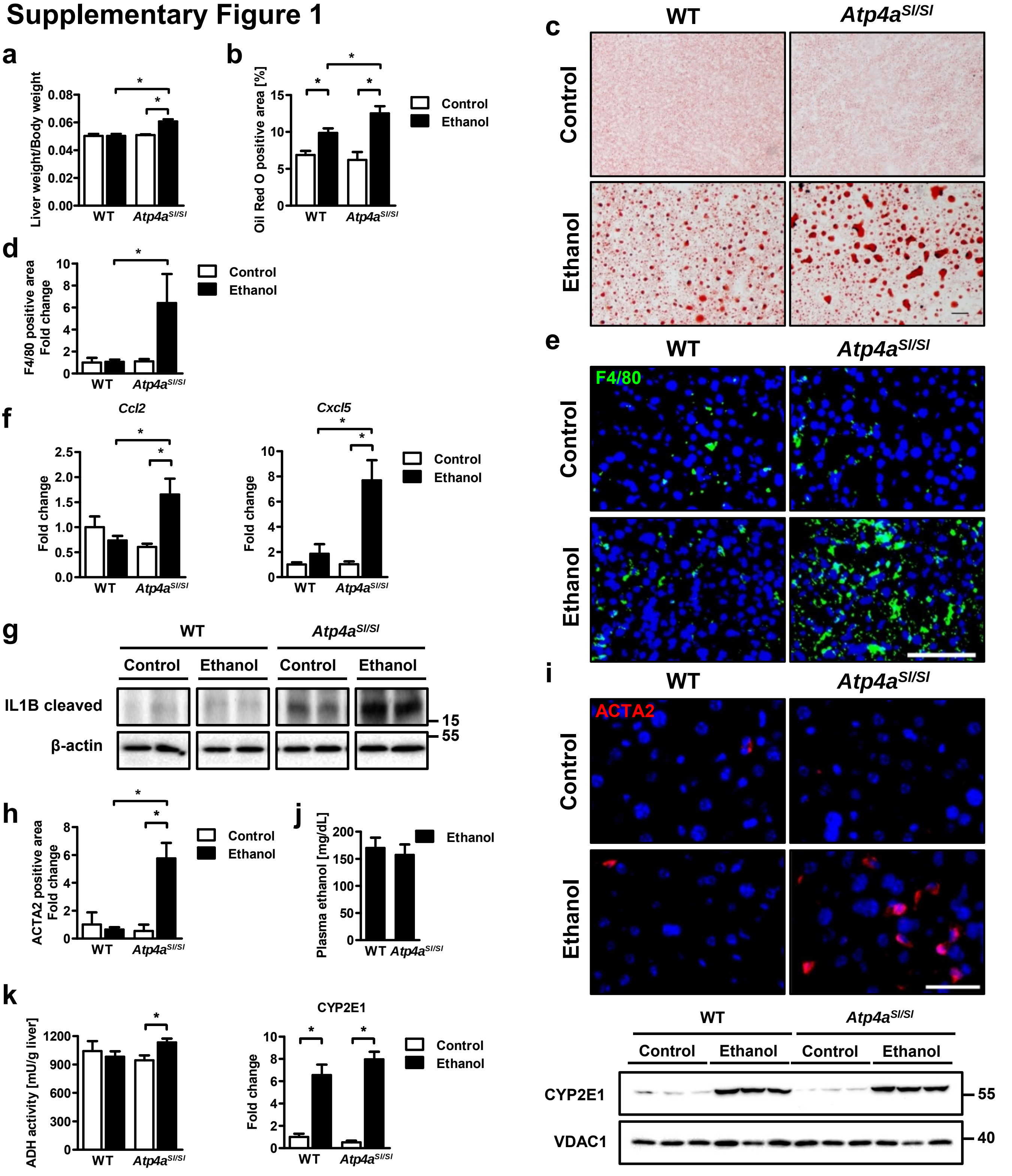

Supplementary Figure 1. *Atp4a$^{Sl/Sl}$* mice are more susceptible to alcohol-induced liver disease. WT mice and their *Atp4a$^{Sl/Sl}$* littermates were fed an oral control diet (n=5–7) (2-3 replicates) or ethanol diet (n=9–15) (8-9 replicates) for 9 weeks. (a) Ratio of liver to body weight. (b) Hepatic areas of steatosis were identified by staining with Oil Red O; area was quantified by image analysis software (n=2–6). (c) Representative Oil Red O-stained liver sections. Scale bar=100 µm. (d–e) Representative liver sections of F4/80 immunofluorescence staining; positively stained area was quantified by image analysis software (n=2–7). Scale bar=50 µm. (f) Hepatic expression of *Ccl2* and *Cxcl5.* (g) Hepatic levels of cleaved IL1B. (h–i) Representative liver sections of ACTA2 immunofluorescence staining; the positively stained area was quantified by image analysis software (n=2–6). Scale bar=20 µm. (j) Plasma levels of ethanol were comparable between WT and *Atp4a$^{Sl/Sl}$* mice following 9 weeks of ethanol feeding. (k) ADH and CYP2E1 are main hepatic enzymes that metabolize ethanol and convert it to acetaldehyde. ADH activity and induction of CYP2E1 (n=3) did not differ significantly between WT and *Atp4a$^{Sl/Sl}$* mice after ethanol feeding. Significance was evaluated using the unpaired Student *t* test or Mann-Whitney U-statistic test. Results are expressed as mean ±.s.e.m. *P<0.05.

Supplementary Figure 2

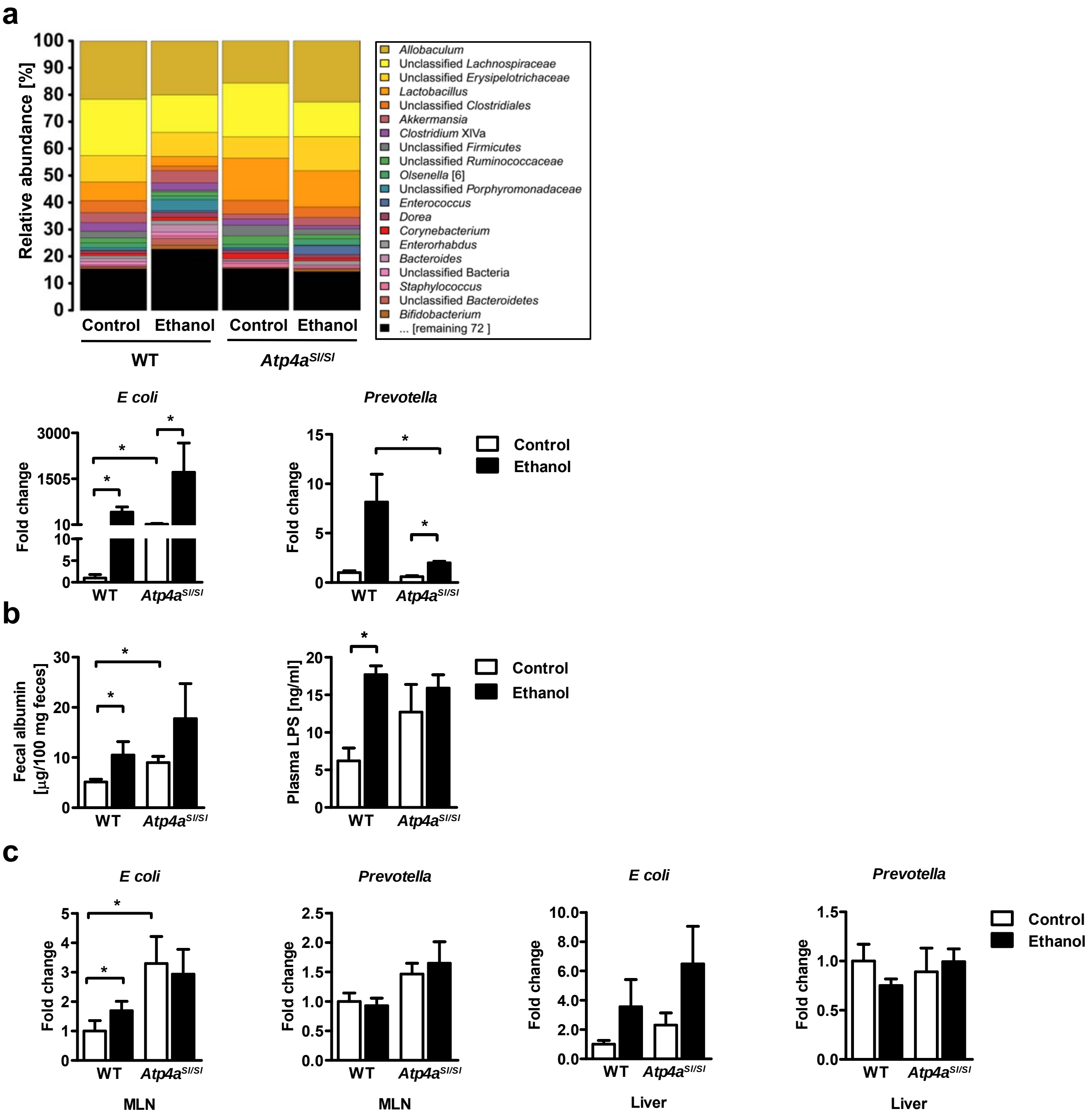

Supplementary Figure 2. Microbiota composition and bacterial translocation in *Atp4a*$^{Sl/Sl}$ mice after chronic alcohol administration. WT mice and their *Atp4a*$^{Sl/Sl}$ littermates were fed an oral control diet (n=3–13) (2-3 replicates) or ethanol diet (n=8–16) (8-9 replicates) for 9 weeks. (a) Fecal samples were collected and 16S rRNA genes were sequenced. The graph demonstrates the relative abundance of each genus (upper panel). Proportion of fecal *E coli* and *Prevotella* were measured by qPCR (lower panels). (b) Paracellular intestinal permeability was evaluated by measuring fecal albumin content and plasma levels of LPS using ELISAs. (c) Numbers of *E coli* and *Prevotella* in mesenteric lymph nodes (MLN) and liver, measured by qPCR. Significance was evaluated using the unpaired Student *t* test or Mann-Whitney U-statistic test. Results are expressed as mean ± s.e.m. **P*<0.05.

Supplementary Figure 3

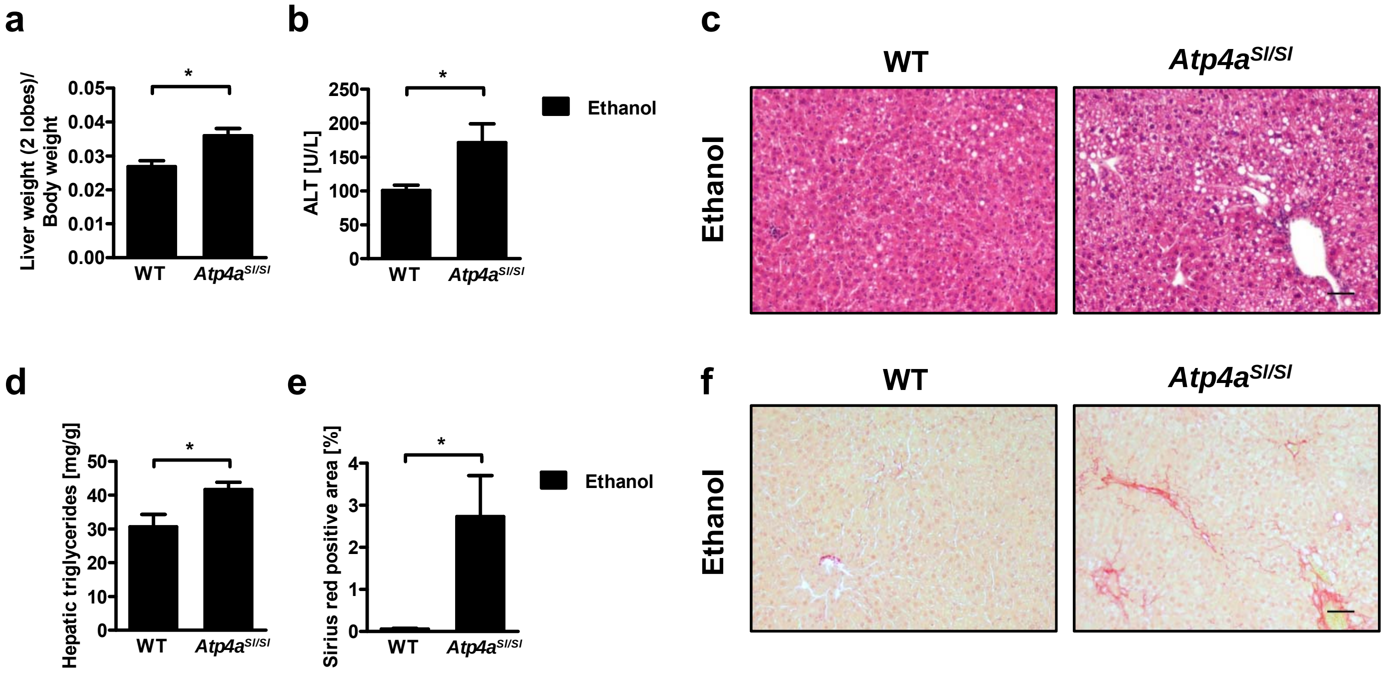

Supplementary Figure 3. Differences in livers of *Atp4a*$^{SI/SI}$ vs WT mice following chronic plus binge-induced alcoholic liver disease. WT mice (n=5–6) and their *Atp4a*$^{SI/SI}$ littermates (n=10–11) were subjected to chronic plus binge ethanol feeding. (a) Ratio of two liver lobes to body weight. (b) Plasma levels of ALT. (c) Representative liver sections after hematoxylin and eosin staining. (d) Hepatic triglyceride content (n=5–7). (e) Hepatic areas of fibrosis were identified by staining with Sirius red; area was quantitated by image analysis software (n=5–6). (f) Representative Sirius red-stained liver sections. Scale bar=100 µm. Significance was evaluated using the unpaired Student *t* test or Mann-Whitney U-statistic test. Results are expressed as mean ± s.e.m. *$P<0.05$.

Supplementary Figure 4

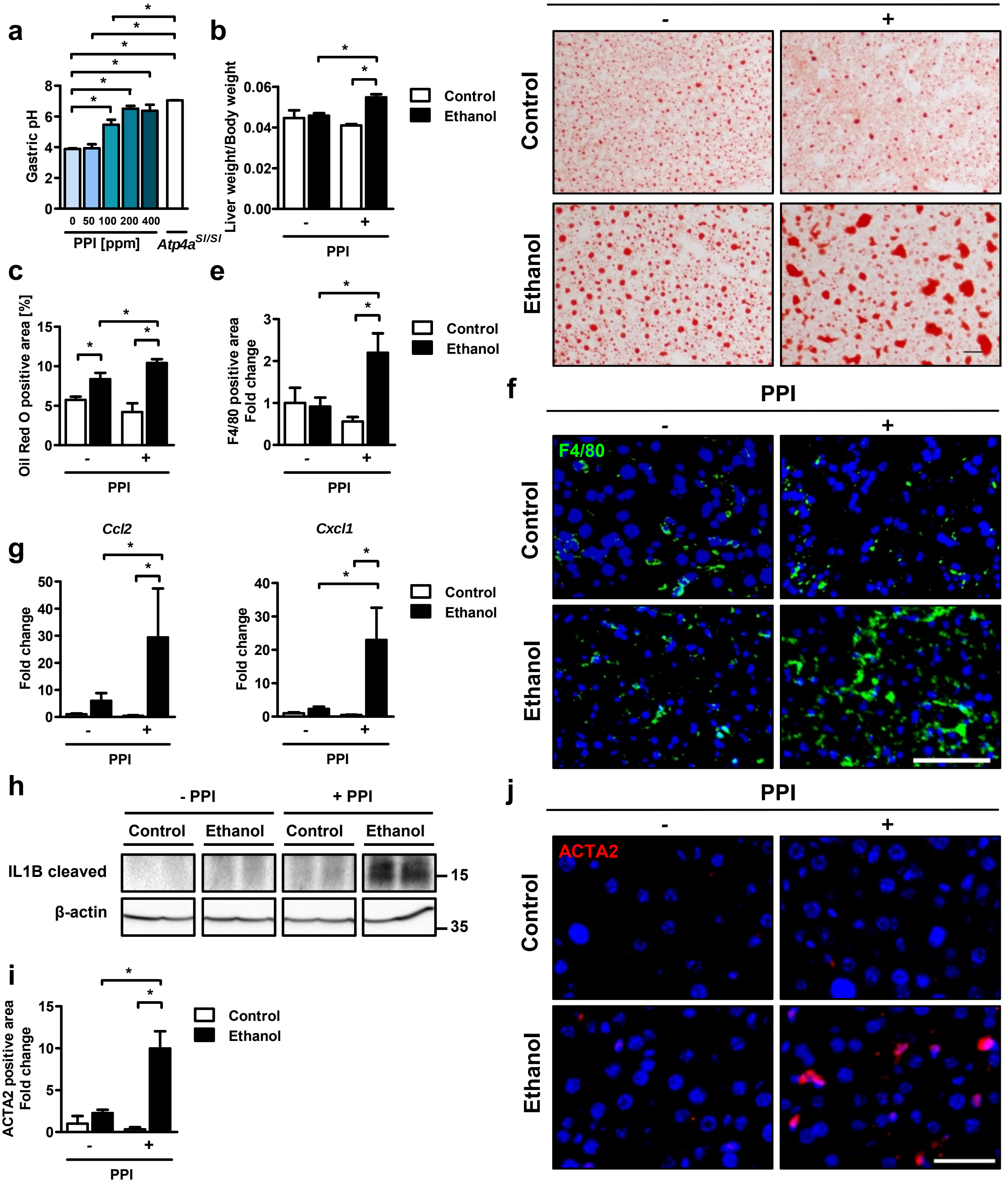

Supplementary Figure 4. PPIs increase alcohol-induced liver disease in mice. (a) C57BL/6 mice were fed an oral control diet containing indicated concentrations of PPI for 4 days (n=5). Gastric pH was measured. For comparison, pH was also measured in the stomach of *Atp4a*$^{Sl/Sl}$ mice (n=4). (b–j) C57BL/6 mice were fed an oral control diet (n=5) (1-2 replicates) or ethanol diet (n=11–13) (1-2 replicates) that contained PPI (200 ppm) or vehicle (water) for 9 weeks. (b) Ratio of liver to body weight. (c) Hepatic areas of steatosis were stained with Oil Red O and quantified by image analysis software (n=4–12). Scale bar=100 µm. (d) Representative Oil Red O-stained liver sections. (e–f) Representative liver sections of F4/80 immunofluorescence staining; the positively stained area was quantified by image analysis software (n=3–9). Scale bar=50 µm. (g) Hepatic expression of *Ccl2* and *Cxcl1.* (h) Hepatic levels of cleaved IL1B. (i–j) Representative liver sections of ACTA2 immunofluorescence staining; the positively stained area was quantified by image analysis software (n=2–6). Scale bar=20 µm. Significance was evaluated using the unpaired Student *t* test or Mann-Whitney U-statistic test. Results are expressed as mean ± s.e.m.. *$P<0.05$.

Supplementary Figure 5

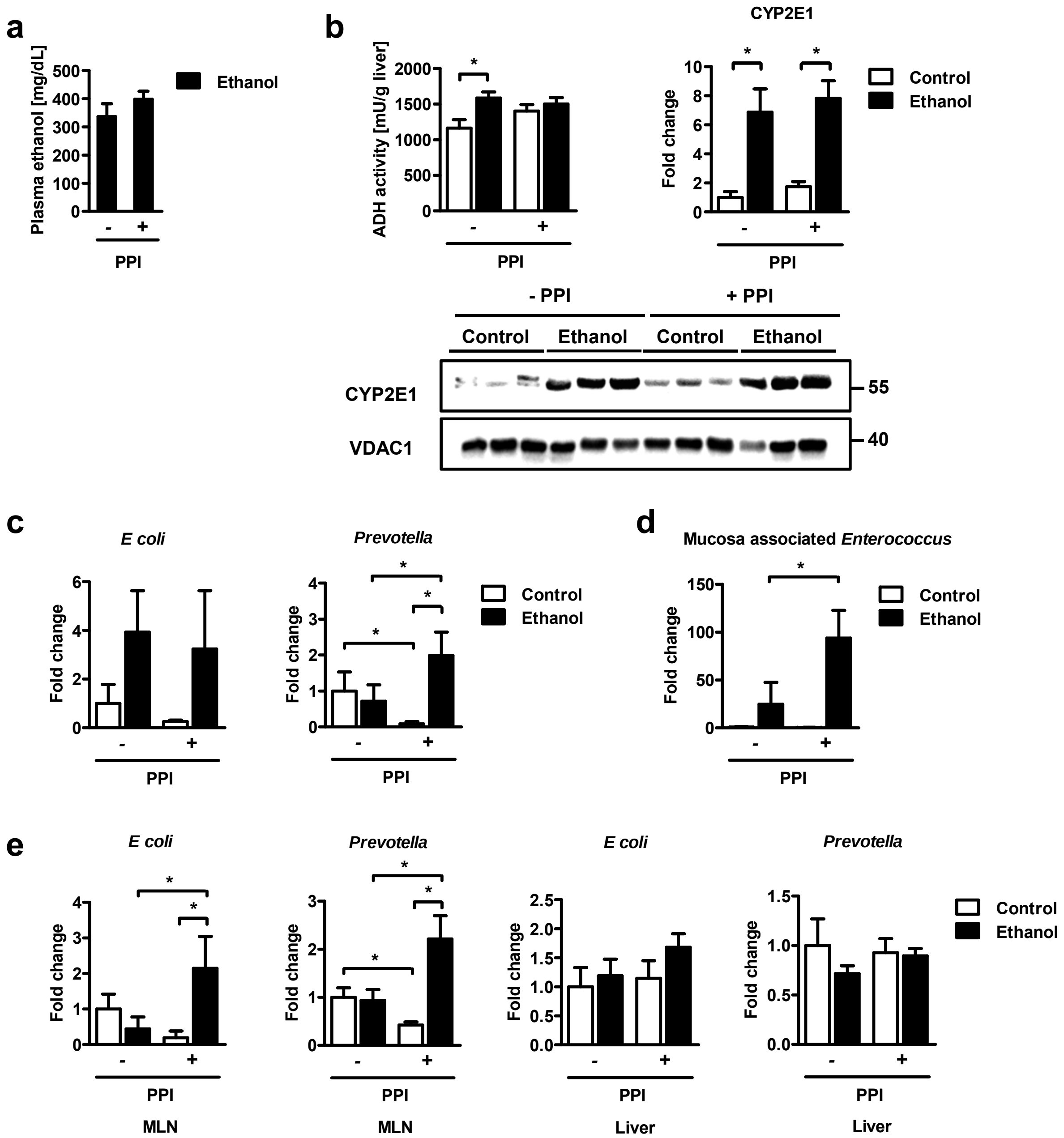

Supplementary Figure 5. Ethanol metabolism, microbiota changes and bacterial translocation in mice given a PPI after chronic alcohol administration. C57BL/6 mice were fed an oral control diet (n=3–5) (1-2 replicates) or ethanol diet (n=5–19) (1-2 replicates) that contained PPI (200 ppm) or vehicle (water) for 9 weeks. (a) Plasma levels of ethanol were comparable between mice fed ethanol for 9 weeks, with or without PPI. (b) Hepatic ADH activity and microsome levels of CYP2E1 protein (n=3) did not differ significantly between mice that were vs were not given PPIs; levels of CYP2E1 increased when mice were fed ethanol. (c) *E coli* and *Prevotella* in fecal samples, measured by qPCR. Proportions of fecal *E coli* did not differ significantly between mice that were vs were not given PPIs. Fecal numbers of *Prevotella* were higher in C57BL/6 mice receiving a PPI than mice not receiving a PPI. (d) *Enterococcus* in the mucosa-associated layer of the small intestine, as assessed by qPCR. (e) *E coli* and *Prevotella* in mesenteric lymph nodes (MLN) and liver, measured by qPCR. Translocation of *E coli* and *Prevotella* to mesenteric lymph nodes, but not to the liver was increased in ethanol-fed mice given a PPI, compared with those that did not receive a PPI. Significance was evaluated using the unpaired Student *t* test or Mann-Whitney U-statistic test. Results are expressed as mean ± s.e.m. **P*<0.05.

Supplementary Figure 6

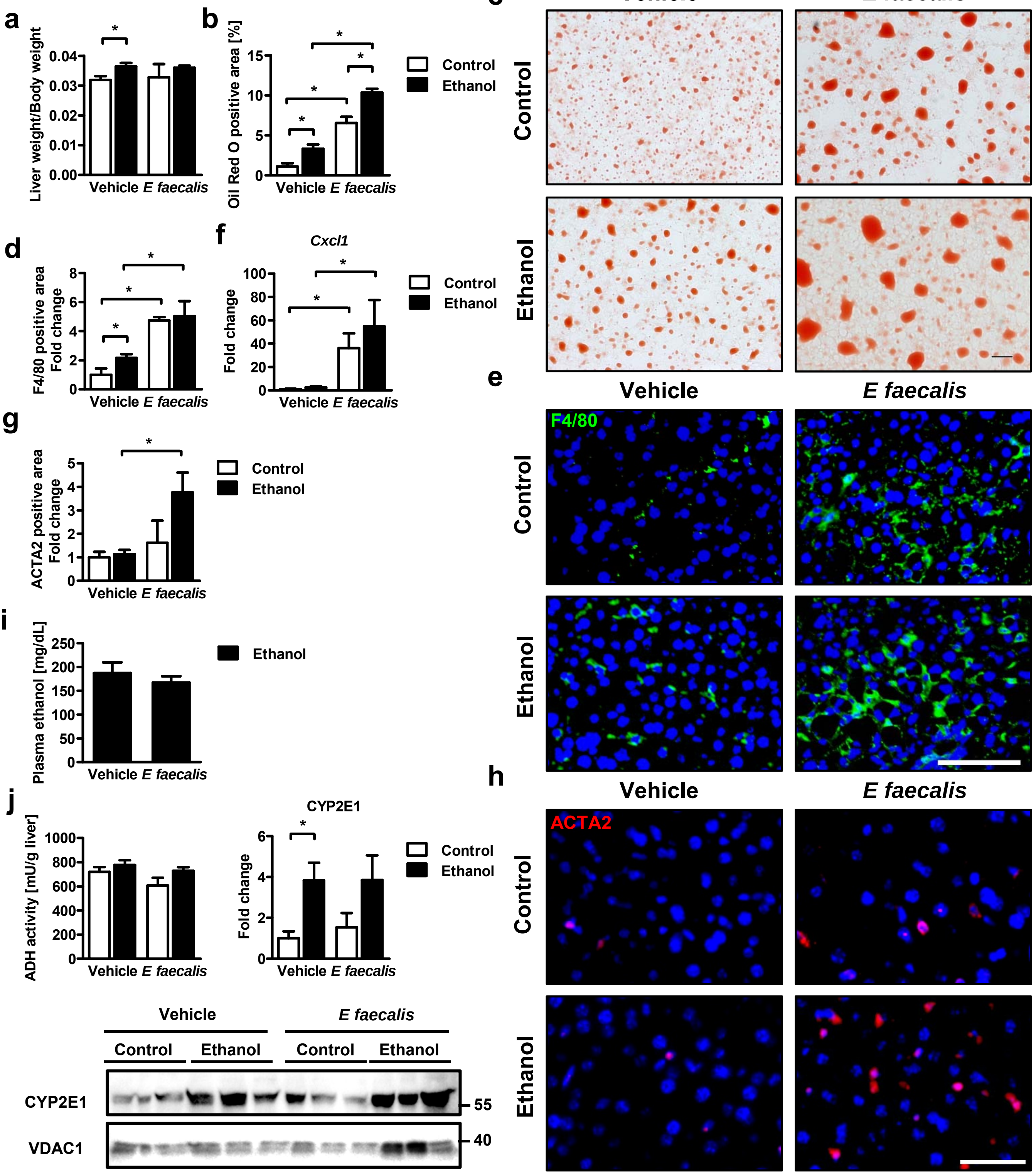

Supplementary Figure 6. *E faecalis* exacerbates ethanol-induced liver disease in mice. C57BL/6 mice were fed an oral control diet (n=4–5) (1-2 replicates) or ethanol diet (n=11–14) (1-2 replicates) for 9 weeks and gavaged with *E faecalis* ($5x10^9$ CFUs) or vehicle (water) every third day. (a) Ratio of liver to body weight. (b) The Oil Red O-stained area was quantified by image analysis (n=3–14). Scale bar=100 µm. (c) Representative Oil Red O-stained liver sections. (d–e) Representative liver sections of F4/80 immunofluorescence staining; the positively stained area was quantified by image analysis software (n=2–10). Scale bar=50 µm. (f) Hepatic gene expression of *Cxcl1*. (g–h) Representative liver sections of ACTA2 immunofluorescence staining; the positively stained area was quantified by image analysis software (n=2–9). Scale bar=20 µm. (i) Plasma levels of ethanol were comparable between mice given the vehicle or *E faecalis* after 9 weeks of ethanol feeding. (j) Hepatic ADH activity and microsome levels of CYP2E1 protein (n=3) did not differ significantly between mice gavaged with vehicle or *E faecalis* following ethanol administration. Significance was evaluated using the unpaired Student *t* test or Mann-Whitney U-statistic test. Results are expressed as mean ± s.e.m. *P<0.05.

Supplementary Figure 7

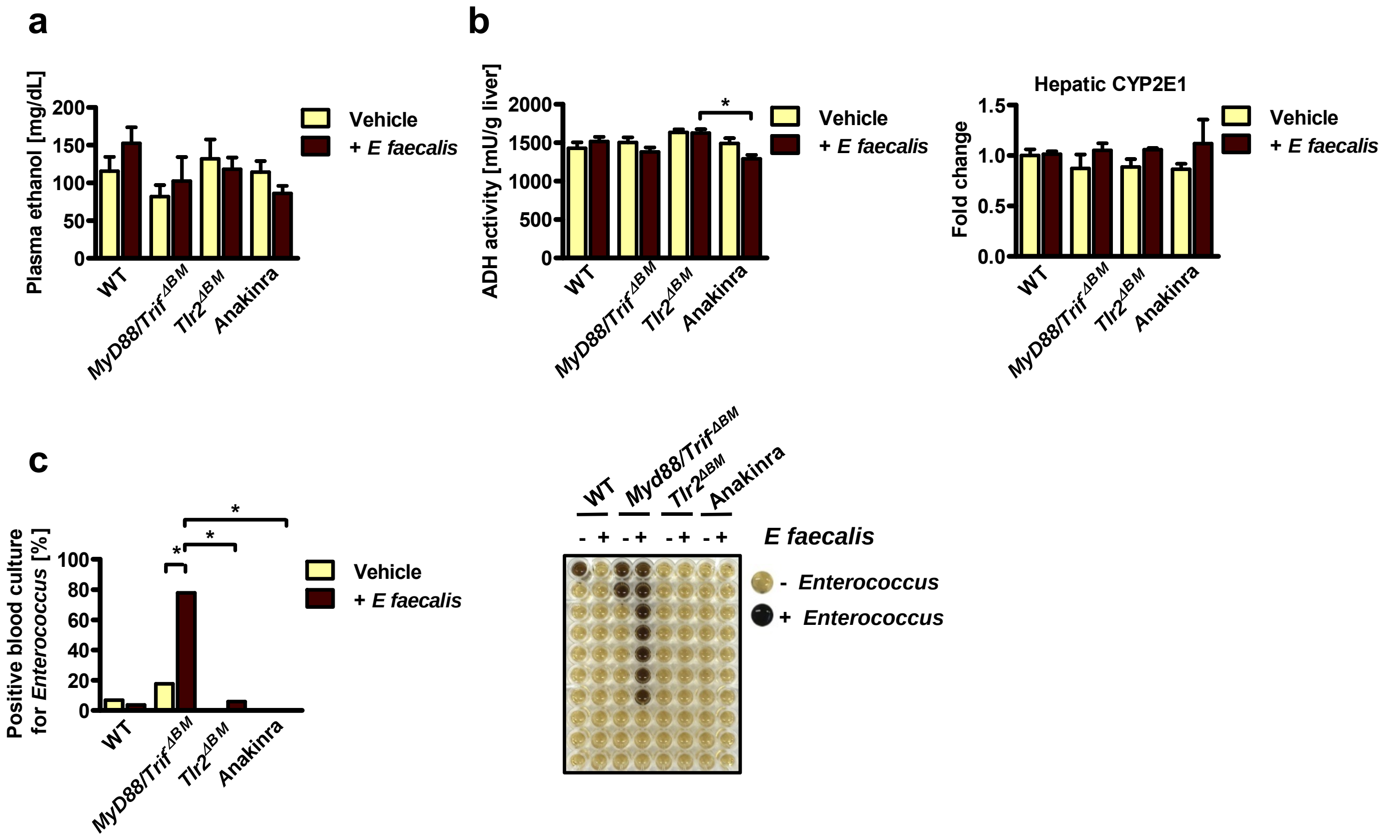

Supplementary Figure 7. Ethanol metabolism and bacterial translocation in bone marrow chimeric mice or treated with an IL1-receptor antagonist after chronic alcohol administration. C57BL/6 mice were irradiated, given transplants of WT, *Myd88$^{-/-}$/Trif$^{LPS2/LPS2}$* (*Myd88/Trif^{DBM}*) or *Tlr2$^{-/-}$* bone marrow (*Tlr2^{DBM}*) and injected with clodronate liposomes. Mice were then gavaged with polymyxin B 150 mg/kg and neomycin 200 mg/kg body weight once daily for 1 week to facilitate colonization of *E faecalis*, fed the ethanol diet for 9 weeks and gavaged with *E faecalis* (5x10^9 CFUs) (n=5–27) (1- 3 replicates) or vehicle (water) (n=5–29) (1-3 replicates) every third day. A subset of WT mice given WT bone marrow transplants received the IL1-receptor antagonist anakinra. (a) Plasma levels of ethanol were comparable between different groups of mice after 9 weeks of ethanol feeding. (b) Hepatic ADH activity and microsome levels of CYP2E1 protein (n=3) were comparable between different groups of mice following ethanol administration. (c) Cultured *Enterococcus* in blood. Representative culture plate. Significance was evaluated using one-way analysis of variance with Newman-Keuls post-test. Fisher's exact test was used in the analysis of positive *Enterococcus* cultures. Results are expressed as mean ± s.e.m. *P<0.05.

Supplementary Figure 8

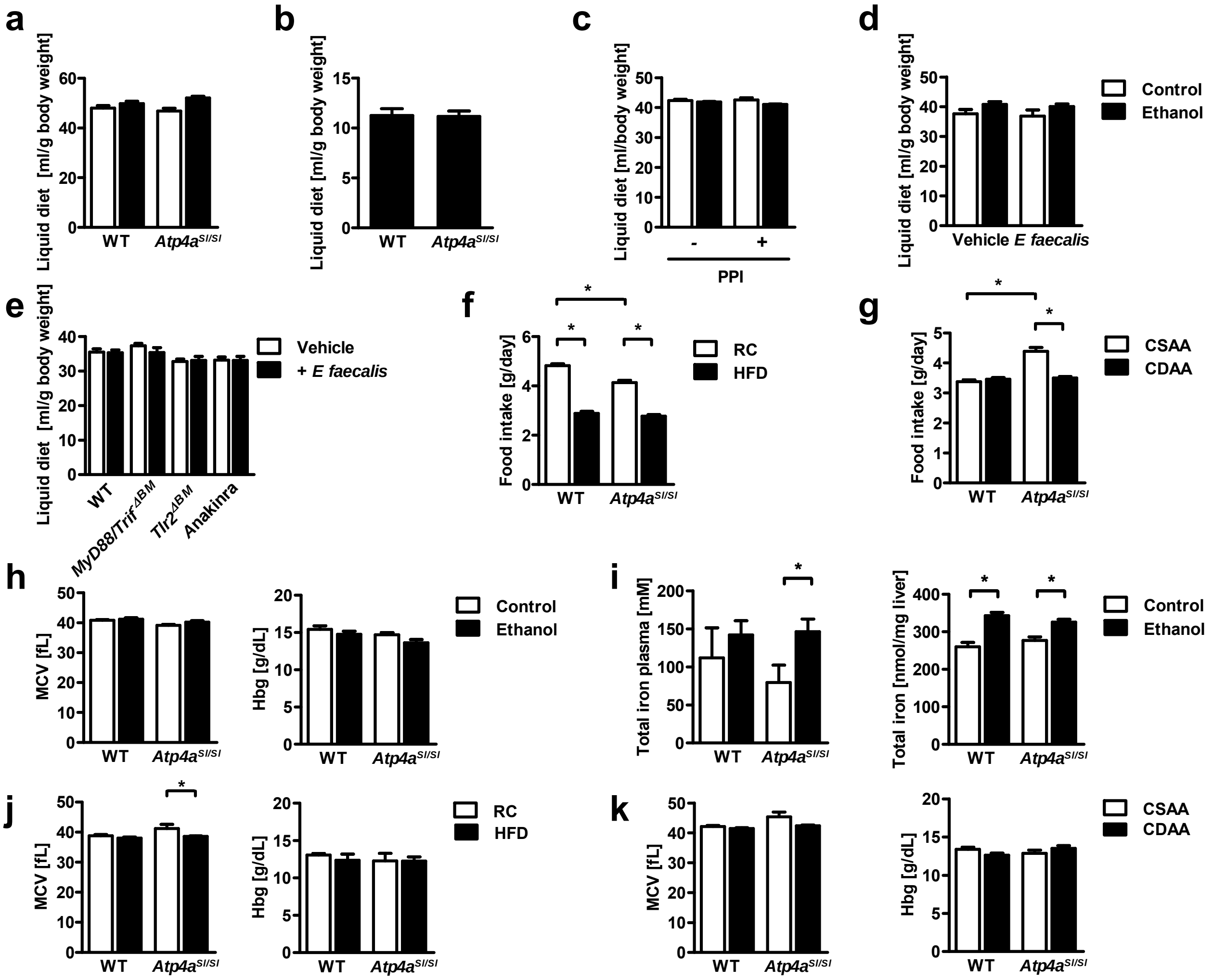

Supplementary Figure 8. Food intake and iron repletion in *Atp4a^{Sl/Sl}* mice. (a) WT mice and their *Atp4a^{Sl/Sl}* littermates were fed a control diet (n=5–7) or an ethanol diet (n=10–14) for 9 weeks. Liquid diet consumption. (b) WT mice (n=6) and their *Atp4a^{Sl/Sl}* littermates (n=11) were subjected to chronic-plus-binge ethanol feeding. Liquid diet consumption. (c) C57BL/6 mice were fed an oral control diet (n=5) or an ethanol diet (n=12–13), which contained PPI (200ppm) or vehicle (water) for 9 weeks. Liquid diet consumption. (d) C57BL/6 mice were fed a control diet or an ethanol diet and gavaged with vehicle (n=5–13) or *E faecalis* ($5x10^9$ CFUs) (n=4–14) every third day for 9 weeks. Liquid diet consumption. (e) C57BL/6 mice were given transplants of WT, *Myd88^{-/-}/Trif^{LPS2/LPS2}* (*Myd88/Trif^{DBM}*) or *Tlr2^{-/-}* bone marrow (*Tlr2^{DBM}*), injected with clodronate liposomes, treated with polymyxin B 150 mg kg^{-1} and neomycin 200 mg kg^{-1} body weight once daily for 1 week, fed ethanol diet for 9 weeks, and gavaged with *E faecalis* ($5x10^9$ CFUs) (n=14–27) or vehicle (water) (n=15–29) every third day. A subset of WT mice given WT bone marrow transplants received the IL1-receptor antagonist anakinra. Liquid diet consumption. (f) WT mice and their *Atp4a^{Sl/Sl}* littermates were fed a RC diet (n=6–8) or a HFD (n=14–15). Food intake was measured during 9 weeks of feeding. (g) WT and *Atp4a^{Sl/Sl}* mice were fed a CSAA (control, n=5–9) or CDAA diet (n=10–12); food intake was measured during 20 weeks of feeding. (h–k) Gastric acid is required for iron solubilization and absorption in the proximal small intestine [1]. *Atp4a^{Sl/Sl}* mice develop iron-deficiency anemia, which can be reversed by 4 weeks of a high-iron diet [1]. At 4 weeks of age, WT and *Atp4a^{Sl/Sl}* mice were fed a high-iron diet for 1 month. Mice were switched to a RC diet for 1 week followed by induction of liver disease. Hemoglobin (Hbg), mean corpuscular volume (MCV), and iron indices were measured following iron repletion and completion of the experiment. (h–i) WT mice and their *Atp4a^{Sl/Sl}* littermates were fed an oral control diet (n=5–7) or ethanol diet (n=9–15) for 9 weeks. There were no significant differences in Hbg, MCV, plasma, or liver iron between WT and *Atp4a^{Sl/Sl}* mice at the end of the treatment period. (j) WT and *Atp4a^{Sl/Sl}* mice were fed a RC (n=5) diet or HFD (n=7–10) for 9 weeks. There were no significant differences in concentrations of Hbg or MCV between WT and *Atp4a^{Sl/Sl}* mice at the end of the treatment period. (k) WT and *Atp4a^{Sl/Sl}* mice were fed a CSAA (control, n=5–7) or CDAA diet (n=9–10) for 20 weeks. There were no significant differences in concentrations of Hbg or MCV between WT and *Atp4a^{Sl/Sl}* mice at the end of the treatment period. Significance was evaluated using either the unpaired Student *t* test or Mann-Whitney U-statistic test. Results are expressed as mean ± s.e.m. $^{*}P<0.05$.

Supplementary Figure 9. Uncropped western blots.

Supplementary Figure 1g

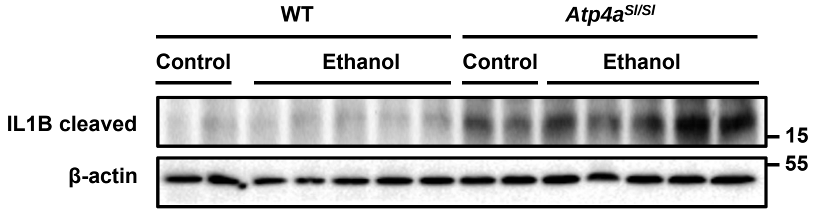

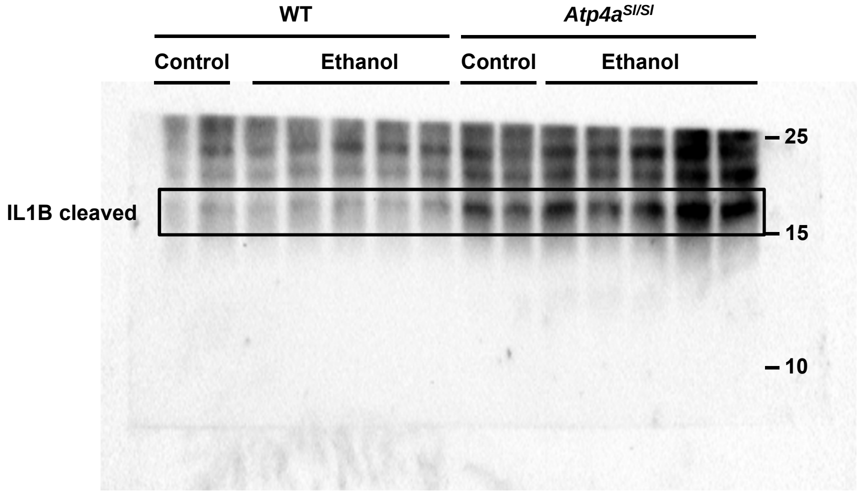

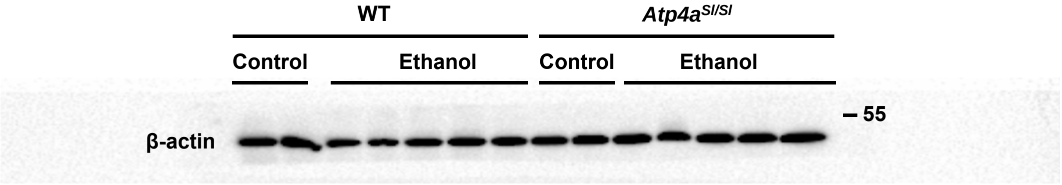

Supplementary Figure 9 (continued)

Supplementary Figure 1k

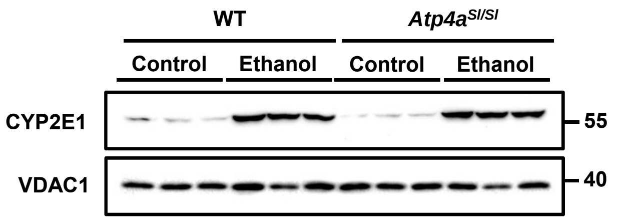

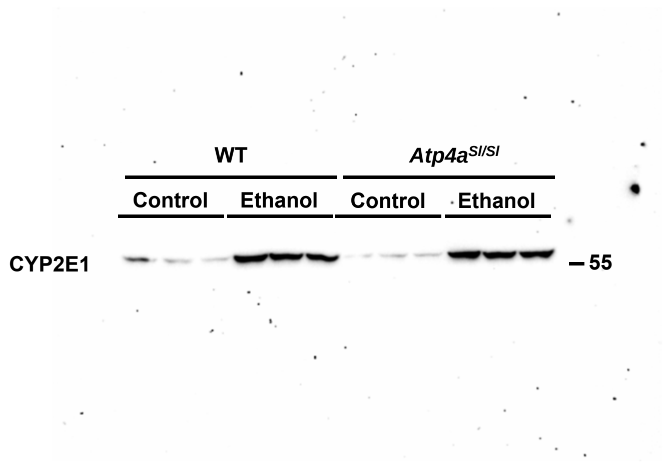

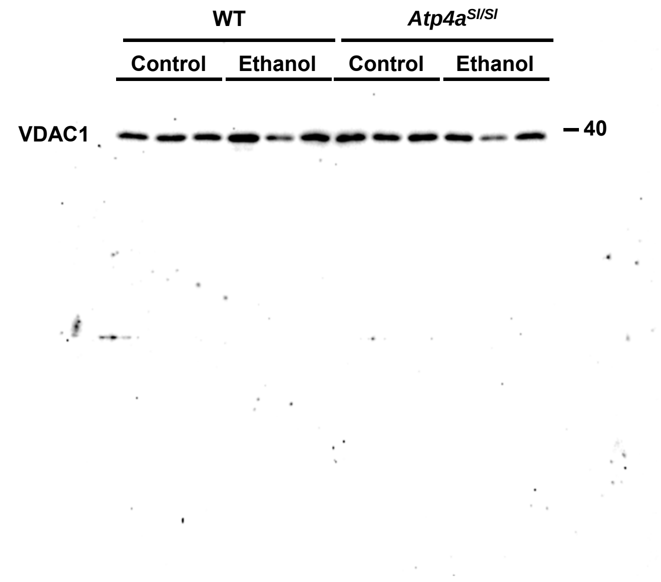

Supplementary Figure 10. Uncropped western blots**.**

Supplementary Figure 4h

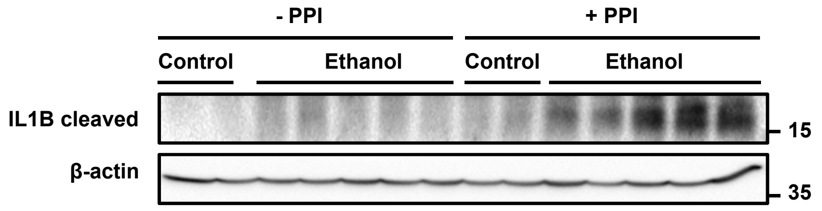

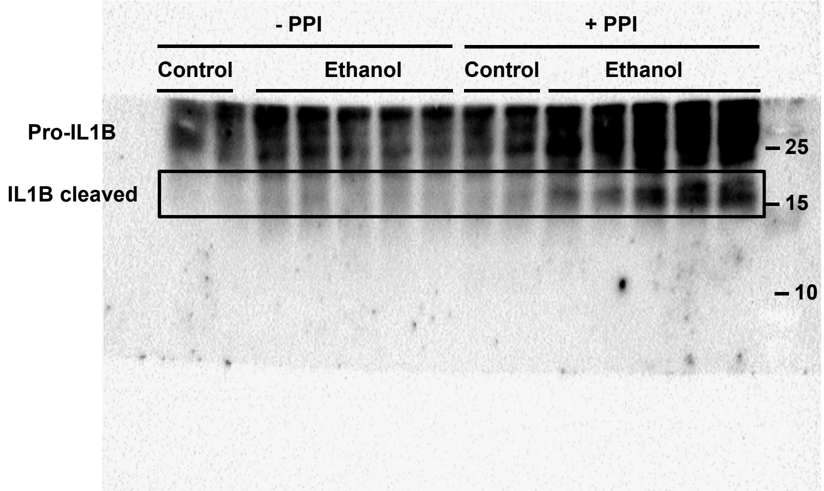

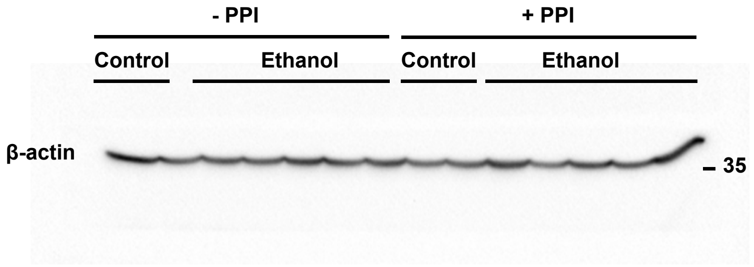

Supplementary Figure 11. Uncropped western blots.

Supplementary Figure 5b

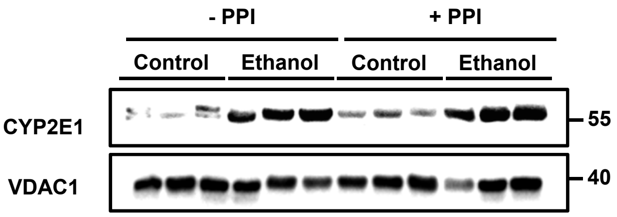

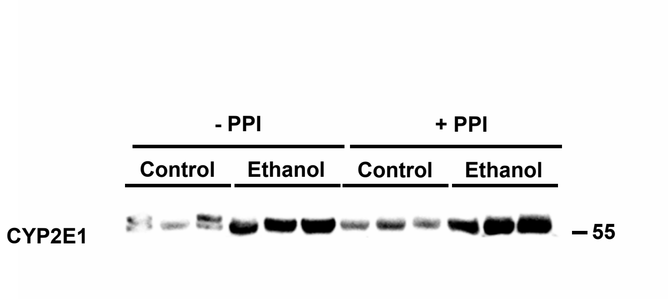

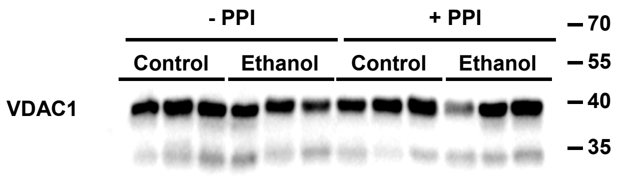

Supplementary Figure 12. Uncropped western blots.

Supplementary figure 6j

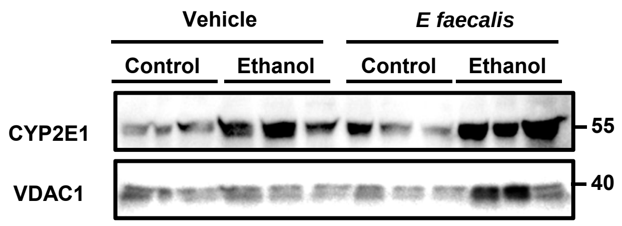

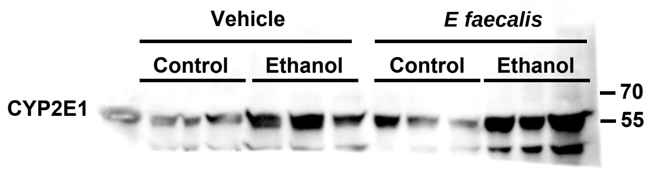

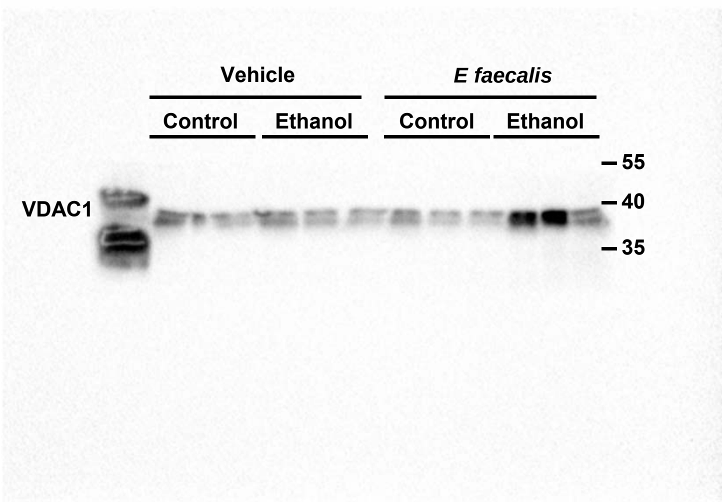

Supplementary Table 1
Characteristics of Study Subjects at Time of Inclusion

	Active users of PPIs	Previous users of PPIs	Never users of PPIs
Number of patients	1024	745	3061
Men (%)	672 (66%)	514 (69%)	2,211 (72%)
Age (years), median (IQR)	58 (49–67)	54 (45–64)	54 (43–63)
Albumin [g L^{-1}], median (IQR)	38 (33–42)	40 (35–44)	40 (35–43)
ALT [U L^{-1}], median (IQR)	35 (21–60)	37 (22–63)	37 (23–69)
Bilirubin [umol L^{-1}], median (IQR)	9 (6–16)	9 (5–15)	10 (6–16)
INR, median (IQR)	1.0 (1.0–1.2)	1.0 (1.0–1.1)	1.0 (1.0–1.2)
Platelets [10^9 L^{-1}], median (IQR)	251 (175–323)	230 (163–302)	230 (168–296)
Creatinine [umol L^{-1}], median (IQR)	68 (55–87)	65 (56–80)	67 (56–82)
Sodium [mmol L^{-1}], median (IQR)	137 (132–140)	138 (135–141)	138 (134–141)

IQR, interquartile range

Supplementary References

1. Krieg, L., *et al.* Mutation of the gastric hydrogen-potassium ATPase alpha subunit causes iron-deficiency anemia in mice. *Blood* **118**, 6418-6425 (2011).
